# Variant‐specific and reciprocal Hsp40 functions in Hsp104‐mediated prion elimination

**DOI:** 10.1111/mmi.13966

**Published:** 2018-04-30

**Authors:** Michael T. Astor, Erina Kamiya, Zachary A. Sporn, Scott E. Berger, Justin K. Hines

**Affiliations:** ^1^ Department of Chemistry Lafayette College Easton PA USA; ^2^ Present address: Geisinger Commonwealth School of Medicine Scranton PA USA

## Abstract

The amyloid‐based prions of *Saccharomyces cerevisiae* are heritable aggregates of misfolded proteins, passed to daughter cells following fragmentation by molecular chaperones including the J‐protein Sis1, Hsp70 and Hsp104. Overexpression of Hsp104 efficiently cures cell populations of the prion [*PSI* ^+^] by an alternative Sis1‐dependent mechanism that is currently the subject of significant debate. Here, we broadly investigate the role of J‐proteins in this process by determining the impact of amyloid polymorphisms (prion variants) on the ability of well‐studied Sis1 constructs to compensate for Sis1 and ask whether any other *S. cerevisiae* cytosolic J‐proteins are also required for this process. Our comprehensive screen, examining all 13 members of the yeast cytosolic/nuclear J‐protein complement, uncovered significant variant‐dependent genetic evidence for a role of Apj1 (antiprion DnaJ) in this process. For strong, but not weak [*PSI* ^+^] variants, depletion of Apj1 inhibits Hsp104‐mediated curing. Overexpression of either Apj1 or Sis1 enhances curing, while overexpression of Ydj1 completely blocks it. We also demonstrated that Sis1 was the only J‐protein necessary for the propagation of at least two weak [*PSI* ^+^] variants and no J‐protein alteration, or even combination of alterations, affected the curing of weak [*PSI* ^+^] variants, suggesting the possibility of biochemically distinct, variant‐specific Hsp104‐mediated curing mechanisms.

## Introduction

Most yeast prions are heritable amyloid aggregates of misfolded proteins (Wickner, 1994; Liebman and Chernoff, 2012). Of the at least 10 amyloid‐forming prions identified to date in the brewer's yeast *Saccharomyces cerevisiae*, the most studied and best‐understood is [*PSI* ^+^], an amyloid aggregate of the translation termination factor Sup35 (Wickner *et al*., 1995; Liebman and Chernoff, 2012). Prion propagation in cell populations requires the action of molecular chaperone proteins, namely Hsp40s (called J‐proteins due to homology to bacterial DnaJ), Hsp70 and Hsp104 (Chernoff *et al*., 1995; Sondheimer *et al*., 2001; Song *et al*., 2005; Higurashi *et al*., 2008). Through the combined action of these chaperone proteins, prions are severed to create additional seeds, called propagons, which can then be inherited to daughter cells during mitosis (Cox *et al*., 2003; Aron *et al*., 2007; Liebman and Chernoff, 2012). Central to this process, the disaggregase Hsp104 has been the subject of intrigue for more than two decades (Chernoff *et al*., 1995). Hsp104 is absolutely required for prion propagation, as depletion, inhibition, or mutation of Hsp104 results in prion loss (Chernoff *et al*., 1995; Jung *et al*., 2002; Eaglestone *et al*., 2000). The Hsp104 disaggregase has six tubular subunits surrounding a central pore with a total of 12 Walker‐type ATPases (Mogk and Bukau, 2004). The currently accepted model of prion fragmentation posits that Hsp104 binds individual polypeptides of amyloid aggregates in a J‐protein and Hsp70‐dependent manner and translocates the protein through the central pore at the expense of ATP (Haslberger *et al*., 2008; Tipton *et al*., 2008; Winkler *et al*., 2012).

Prions can adopt distinct amyloid structures (amyloid structural polymorphisms), called ‘strains’ in mammalian systems and ‘variants’ in yeast, that dictate the intensity of yeast prion‐associated phenotypes and stability in mitosis. Prion variants are numerous and diverse but are typically referred to as ‘strong’ or ‘weak’, referring to phenotypic strength, which often correlates to mitotic stability. For example, in the case of [*PSI* ^+^], weak variants tend to have larger amyloid fibers with fewer free ends, resulting in fewer transmissible aggregates to propagate the prion as well as a greater amount of soluble Sup35 (Liebman and Chernoff, 2012). Prion variants often have distinct requirements of chaperone activity, particularly with respect to J‐protein activity, for stable propagation (Derkatch *et al*., 1996; Hines *et al*., 2011a, Prusiner, 2013; Stein and True, 2014a,b; Harris *et al*., 2014; Sporn and Hines, 2015; Schilke *et al*., 2017; Killian and Hines, 2018). The J‐protein Sis1 is specifically required for the propagation of at least four yeast prions and has been shown to be the sole cytosolic J‐protein required for the propagation of strong [*PSI* ^+^] variants (Sondheimer *et al*., 2001; Higurashi *et al*., 2008; Tipton *et al*., 2008; Hines *et al*., 2011bb; Schilke *et al*., 2017).

**Figure 1:**
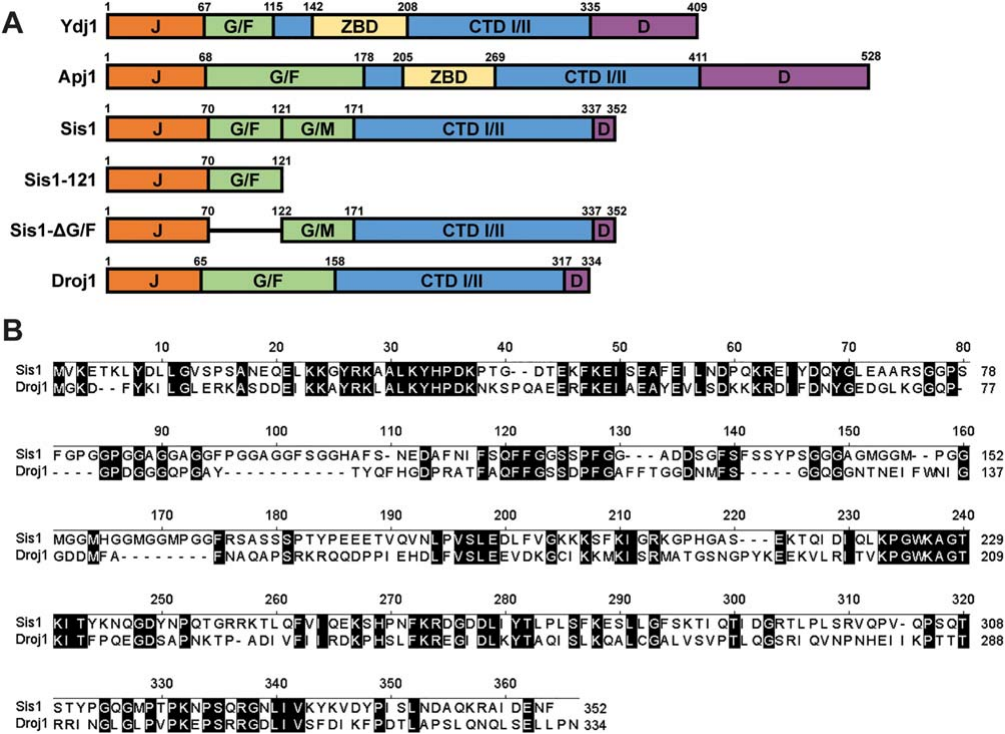
J‐protein primary structure diagrams and Sis1/Droj1 sequence alignment. A. Comparison of primary structures of J‐proteins and J‐protein constructs used in this study. Protein regions are denoted using the following notation: J, J‐domain; G/F, glycine/phenylalanine‐rich region; G/M, glycine/methionine‐rich region; ZBD, zinc binding domain, CTD I/II, C‐terminal peptide‐binding domains I and II; D, dimerization domain. Lines indicate where a region has been deleted. B. Primary sequence alignment between Sis1 (top) and Droj1 (bottom) using the Jotun Hein algorithm of MegAlign from DNAstar (DNASTAR, Madison, WI). Identical residues are highlighted. Numbers above the sequence refer to residue positions in the consensus sequence; numbers to the right indicate residues in each protein.

J‐proteins are obligate co‐chaperones of Hsp70s and act to stimulate Hsp70 ATPase activity, which in turn enhances Hsp70 client peptide binding (Kampinga and Craig, 2010). Nucleotide exchange factors complete the Hsp70 functional cycle by stimulating ADP release, allowing Hsp70 to repeatedly bind and release client polypeptides to accomplish protein refolding and translocation among other myriad tasks (see Craig and Marszalek, 2017 for recent review) (Craig and Marszalek, 2017). Some J‐proteins can also bind polypeptides directly and deliver them to Hsp70s. Thus, J‐proteins can act as specificity factors, directing and diversifying Hsp70 function. Current models posit that Sis1 and the cytosolic yeast Hsp70 Ssa are essential to yeast prion fragmentation by working in unison upstream of Hsp104 to potentially expose or otherwise activate a polypeptide segment of the amyloid aggregate and to recruit Hsp104 in a productive manner to prion aggregates (Aron *et al*., 2007; Higurashi *et al*., 2008; Tipton *et al*., 2008; Winkler *et al*., 2012). In addition to Sis1, 12 other J‐proteins at least partially co‐inhabit the yeast cytosol with prion aggregates (Sahi and Craig, 2007). Of these, three have been previously implicated in prion biology: Ydj1, Swa2 and Apj1 (Bradley *et al*., 2002; Kryndushkin *et al*., 2002; Lian *et al*., 2007; Hines *et al*., 2011b; Hines and Craig, 2011; Troisi *et al*., 2015). Ydj1 is the most abundant J‐protein in the yeast cytosol (Ghaemmaghami *et al*., 2003) and is necessary for propagation of the [*SWI* ^+^] prion (Hines *et al*., 2011b), whereas Swa2, the yeast homolog of mammalian auxilin, was recently found to be essential for the propagation of the [*URE3*] (Troisi *et al*., 2015; Oliver *et al*., 2017). Apj1, which has a primary structure most resembling Ydj1 (Fig. 1A), was initially identified as a factor capable of curing a synthetic prion when overexpressed (Kryndushkin *et al*., 2002) and plays a critical role in the degradation of sumoylated proteins (Sahi *et al*., 2013).

The induction of Hsp104 following exposure of cells to environmental stressors, such as heat, ethanol and ultraviolet light, originally revealed chaperone functions of Hsp104 that promote the disaggregation of otherwise intractable protein aggregates, followed by the discovery that ectopic overexpression of Hsp104 efficiently cures yeast cell populations of [*PSI* ^+^] (Chernoff *et al*., 1995; Derkatch *et al*., 1997). Due to this antiamyloid effect, ectopic expression of Hsp104 has been proposed as a therapeutic intervention against neurodegenerative diseases (Vashist *et al*., 2010; Shorter, 2011). Initially, [*PSI* ^+^] elimination was interpreted as the disaggregation of the prion ‘template’ due simply to an overabundance of Hsp104's disaggregase activity (Chernoff *et al*., 1995; Paushkin *et al*., 1996). Several lines of evidence have pointed away from this simple model of a single, but overactive, function for Hsp104. For example, despite Hsp104's central role in the fragmentation of multiple prions, ectopic Hsp104 overexpression efficiently cures [*PSI* ^+^] but no other prions (Derkatch *et al*., 1997; Moriyama *et al*., 2000; Volkov *et al*., 2002; Du *et al*., 2008; Patel *et al*., 2009; Saifitdinova *et al*., 2010; Holmes *et al*., 2013), begging the question as to how prion‐specificity in this process is manifested if curing occurs by the same mechanism as fragmentation. Additionally, the observation that [*PSI* ^+^] aggregates, as resolved in agarose gels under semi‐denaturing conditions, increase in size prior to elimination by Hsp104 overexpression was equally enigmatic (Kryndushkin *et al*., 2003). Finally, Hsp104 lacking its N‐terminal domain is able to propagate [*PSI* ^+^] and function in prion aggregate dissolution via thermotolerance but does not cure [*PSI* ^+^] when overexpressed, indicating that the biochemical mechanism of aggregate fragmentation to produce propagons is likely distinct from the mechanism of curing by Hsp104 overexpression (Hung and Masison, 2006).

To date, the mechanism by which Hsp104 cures [*PSI* ^+^] specifically remains the subject of significant debate in literature as numerous distinct models have been proposed (Winkler *et al*., 2012; Helsen and Glover, 2012b, Park *et al*., 2014; Ness *et al*., 2017; Zhao *et al*., 2017; Cox and Tuite, 2018; Greene *et al*., 2018; Matveenko *et al*., 2018). In just the past year, significant evidence for two, largely conflicting models has been presented (Ness *et al*., 2017; Zhao *et al*., 2017). One asserts that Hsp104 cures [*PSI* ^+^] by causing malpartitioning of propagons during cell division (Liebman and Chernoff, 2012; Ness *et al*., 2017; Cox and Tuite, 2018; Matveenko *et al*., 2018). Malpartitioning is proposed to occur due to Hsp70‐independent binding of Hsp104 to [*PSI* ^+^] aggregates, followed by the anchoring of aggregates to a cellular structure (likely a cytoskeletal element) in a process that requires Hsp104 ATPase activity (Ness *et al*., 2017; Cox and Tuite, 2018). A second model posits that Hsp104 has additional functionality, distinct from aggregate fragmentation, termed ‘trimming’, in which Hsp104 removes Sup35 monomers from the ends of the prion fibrils and dissolves them when the remaining prion core is presented to the proteasome (Park *et al*., 2014; Zhao *et al*., 2017; Greene *et al*., 2018). This model posits that trimming normally occurs at a low rate and thus has a negligible impact on prion propagation, but becomes relevant upon ectopic overexpression of Hsp104 (Park *et al*., 2014). Although these models provide a potential basis for understanding Hsp104‐mediated [*PSI* ^+^] curing, neither explicitly addresses the role of J‐proteins in Hsp104‐mediated curing.

In addition to its role in prion fragmentation for stable propagation, Sis1 is essential for [*PSI* ^+^] curing by Hsp104 overexpression, as depletion or mutation of Sis1 antagonizes curing whereas Sis1 overexpression accelerates it (Kryndushkin *et al*., 2011; Kirkland *et al*., 2011; Newnam *et al*., 2011; Kiktev *et al*., 2012; Sporn and Hines, 2015). However, despite significant work on the role of Sis1 in prion propagation, its role in [*PSI* ^+^] curing via Hsp104 overabundance is much less clear. As shown in Fig. 1A, Sis1 lacks the characteristic Zn‐binding domain of Ydj1 and other Type I J‐proteins, instead having an archetypical Type II domain architecture which includes an N‐terminal J‐domain, glycine‐rich regions rich in phenylalanine (G/F) and methionine (G/M), two C‐terminal beta‐barrel peptide binding domains (CTD I and II) and a C‐terminal dimerization domain (D). Deletion of either the glycine‐phenylalanine‐rich (G/F) region (Fig. 1A) or the dimerization domain of Sis1 drastically inhibits Hsp104 curing (Kirkland *et al*., 2011). Recently we found that the human ortholog Hdj1 (DNAJB1) can replace Sis1 in the propagation of strong [*PSI* ^+^] variants but is deficient in complementing Sis1's unknown role in Hsp104‐mediated curing, a property that may arise from distinctions between the glycine‐rich regions of these proteins (Sporn and Hines, 2015). To date, however, all investigations into the role of Sis1 in this mechanism have utilized only strong variants of [*PSI* ^+^] (Kirkland *et al*., 2011; Kryndushkin *et al*., 2011; Sporn and Hines, 2015) and little is known about the potential involvement of other J‐proteins in this process.

Here, we expand upon prior investigations into the Sis1 requirement for Hsp104‐mediated [*PSI* ^+^] curing, asking whether the *Drosophila melanogaster* ortholog of Sis1, Droj1, can compensate for Sis1, to what extent prion amyloid structure (i.e., variant identity) affects experimental outcomes, and finally if any of the other 12 J‐proteins located in the *S. cerevisiae* cytosol are also necessary for this process. Our investigation revealed that weak [*PSI* ^+^] variants can be maintained in the absence of any of these 12 J‐proteins, ruling out essential roles for any in weak [*PSI* ^+^] prion propagation. Likewise, elimination of these variants by Hsp104 overexpression was ubiquitous, demonstrating that only Sis1 is necessary for Hsp104‐mediated elimination. In sharp contrast, however, we found that strong variants of [*PSI* ^+^] exhibited exceptional resistance to Hsp104‐mediated elimination in strains lacking the J‐protein Apj1. Loss of Apj1 inhibited Hsp104‐mediated [*PSI* ^+^] curing whereas its overexpression enhanced it. Apj1 has been implicated in amyloid biology several times (Hines *et al*., 2011b; Kryndushkin *et al*., 2002), sometimes acting similarly to Ydj1 (Hines *et al*., 2011b, Hines and Craig, 2011; Kryndushkin *et al*., 2002). Here, we identify a novel genetic interaction between Apj1 and prions that is distinct from Ydj1. We found that while Apj1 promotes Hsp104‐mediated curing of strong variants, Ydj1 potently blocks it when overexpressed. Because no J‐protein alteration, individually or in combination, affected the curing of weak [*PSI* ^+^] variants, our accumulated data raise the question of whether Hsp104 cures weak and strong [*PSI* ^+^] variants by distinct mechanisms, only one of which is dependent upon J‐protein activity.

## Results

### The D. melanogaster Sis1 ortholog, Droj1, propagates strong, but not weak, variants of [*PSI* ^+^]

**Figure 2:**
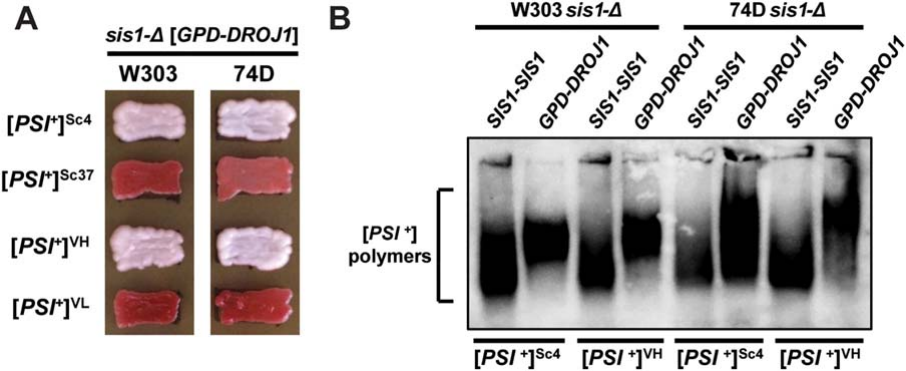
Droj1 supports strong, but not weak, [*PSI^+^*] variants. A. [*PSI* ^+^] cells of the W303 (*left column*) and 74D‐694 (*right column*) genetic backgrounds lacking genomic Sis1 but expressing Sis1 from a *URA3*‐marked plasmid were transformed with a plasmid expressing Droj1 (*GPD‐DROJ1*). Following loss of the *URA3*‐marked plasmid, cells were passaged onto rich medium to test for prion maintenance. Across both backgrounds, four different [*PSI* ^+^] variants, two strong and two weak, were examined. Color phenotype assays are shown for representative transformants (*n* ≥ 12 for each strain). B. Cell lysates of strains bearing strong [*PSI^+^*] variants Sc4 and VH in the W303 and 74D‐694 yeast genetic backgrounds expressing either Sis1 or Droj1 were resolved by SDD‐AGE and subjected to immunoblot analysis using an antibody specific to Sup35.

Sis1 is essential for [*PSI* ^+^] curing by Hsp104 overexpression, as mutation of Sis1 or substitution with some orthologs blocks curing whereas Sis1 overexpression accelerates it (Kirkland *et al*., 2011; Kryndushkin *et al*., 2011; Sporn and Hines, 2015). However, the role Sis1 plays in this process is still unknown. Sis1 is also essential for cell viability. The human ortholog of Sis1, Hdj1, is capable of substituting for Sis1 to maintain cell viability and [*RNQ^+^*] (also sometimes called [*PIN* ^+^]) prion propagation (Lopez *et al*., 2003). Recently, we also demonstrated that Hdj1 is capable of propagating strong but not weak [*PSI* ^+^] variants (Harris *et al*., 2014) but severely deficient in replacing Sis1 in Hsp104‐mediated curing, a characteristic that we speculated may be due to differences in the glycine‐rich regions of the two proteins (Sporn and Hines, 2015). Similar to Hdj1, the *D. melanogaster* ortholog of Sis1, Droj1 (Fig. 1A and B), is also capable of rescuing cell viability (Marchler and Wu, 2001) and maintaining [*RNQ^+^*] (Lopez *et al*., 2003) in a *sis1*‐Δ strain and, most notably, shares many of the same sequence elements as Hdj1. The ability of Droj1 to substitute for Sis1 in [*PSI^+^*] propagation or Hsp104‐mediated curing has never been tested and may provide support for our previous hypothesis regarding Hdj1's deficiency in this process as well as a second higher organism ortholog to compare evolutionarily acquired amino acid changes against Sis1. In order to directly compare Hdj1, Sis1 and Droj1, we examined the relevance of prion variant and yeast genetic background on [*PSI^+^*] propagation in a *sis1‐Δ* strain. To create cells expressing Droj1 in place of Sis1, [*PSI^+^*] *sis1‐Δ* strains expressing Sis1 from a *URA3*‐marked plasmid and harboring one of the four [*PSI* ^+^] variants in the W303 genetic background were used. In these strains, [*psi* ^–^] colonies appear red on rich medium due to a blockage in the adenine biosynthesis pathway, whereas [*PSI* ^+^] colonies appear pink due to [*PSI* ^+^]‐dependent nonsense suppression that allows for partial adenine prototrophy (see *Methods* section for additional details including prion variant origin). These strains were transformed with a multicopy plasmid expressing Droj1 (*GPD‐DROJ1*) and, following selection for the new plasmid, plated onto medium containing 5‐fluoroorotic acid (5‐FOA), which counterselects against the *URA3*‐marked *SIS1* plasmid. Following confirmation of loss of the Sis1‐marked plasmid by uracil auxotrophy and Western blotting (not shown), cells were plated onto rich medium to examine prion maintenance (Fig. 2A, left column). We found that Droj1 is capable of replacing Sis1 only for the propagation of strong [*PSI^+^*] variants; across two experimental trials, we found that in the W303 background, 23/23 maintained [*PSI^+^*]^Sc4^ (strong variant), 0/25 maintained [*PSI^+^*]^Sc37^ (weak variant), 22/22 maintained [*PSI^+^*]^VH^ (strong variant) and 0/22 maintained [*PSI^+^*]^VL^ (weak variant).

Investigations in *S. cerevisiae* are often limited by the use of only a single genetic background for practical purposes, allowing for the possibility that polymorphisms of a particular yeast strain may grossly change experimental outcomes and/or interpretations (Sondheimer *et al*., 2001; Lopez *et al*., 2003; Harris *et al*., 2014; Hines *et al*., 2011a; Kryndushkin *et al*., 2011; Sporn and Hines, 2015). Indeed, incongruencies among the observations of prion–chaperone interactions have been attributable to yeast strain variations multiple times in the past. Specifically in two previous investigations, we uncovered unexpected distinctions in the behavior of some [*PSI* ^+^] variants upon the reduction of Sis1 expression or replacement with Hdj1 between the W303 genetic background and 74D‐694, a second background commonly used in prion investigations (Hines *et al*., 2011a; Sporn and Hines, 2015). These distinctions imply that some unidentified factors that differ between these two backgrounds affect prion‐chaperone interactions in vivo. To ensure that these outcomes are not due to an unknown polymorphism of the W303 background, we utilized a set of [*PSI* ^+^] Sis1‐plasmid shuffling strains in the 74D‐694 background constructed in a previous investigation (Harris *et al*., 2014) and reexamined the Sis1 domain requirements for all of the variants described above. The results were summarily consistent with those obtained in the W303 background, again across two experimental trials in the 74D‐694 background: 23/23 maintained [*PSI^+^*]^Sc4^, 0/24 [*PSI^+^*]^Sc37^, 24/24 maintained [*PSI^+^*]^VH^ and 0/22 maintained [*PSI^+^*]^VL^ (Fig. 2A, right column).

To confirm that colony color accurately reports the maintenance or loss of [*PSI* ^+^] in our strains and to interrogate aggregate size in these strains, we next verified our results using a biochemical assay, semidenaturing detergent agarose gel electrophoresis (SDD‐AGE), in which detergent‐resistant aggregates are resolved using an agarose gel and then visualized by immunoblotting (Kryndushkin *et al*., 2003). In all cases, SDD‐AGE analysis confirmed our colony color observations, however, it also revealed that the substitution of Droj1 for Sis1 in [*PSI^+^*] propagation results in an increase in the size of aggregates resolved in the gel (Fig 2B). This effect was observed universally across both strong [*PSI^+^*] variants and in both yeast genetic backgrounds. This size increase is congruent with the idea that Droj1 can minimally replace Sis1 in prion propagation, but with some loss of functionality, resulting in an apparent partial loss of fragmentation efficiency.

### Droj1 is unable to substitute for Sis1 in the curing of strong [*PSI* ^+^] by Hsp104 overexpression

**Figure 3:**
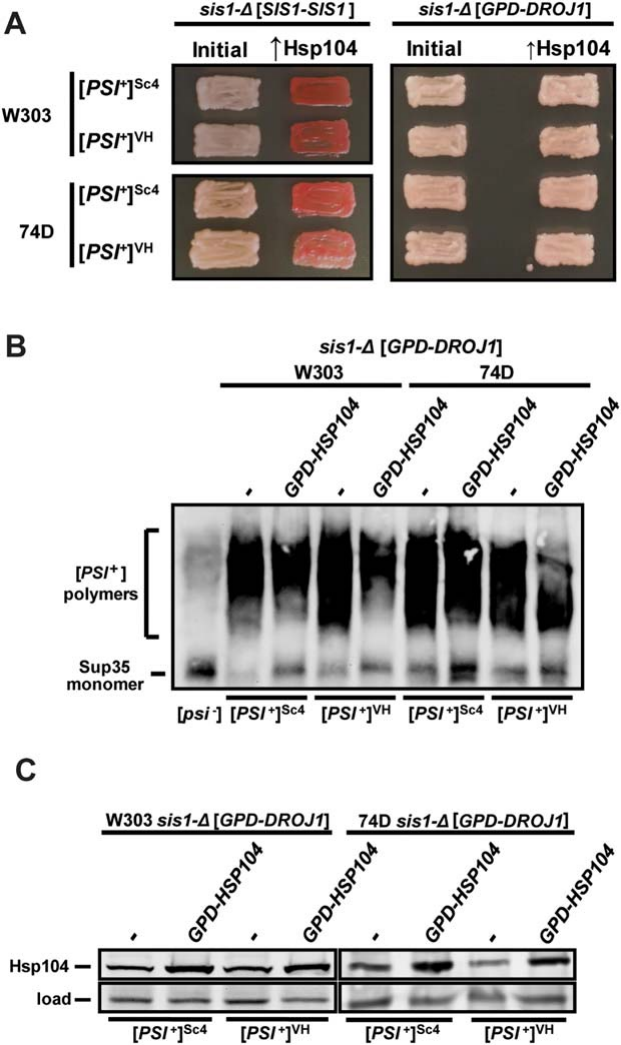
Droj1 is deficient in supporting Hsp104‐mediated [*PSI^+^*] curing. A. Strong [*PSI* ^+^] variants of the W303 (top half) and 74D‐694 (*bottom half*) genetic backgrounds possessing either a plasmid expressing Sis1 (*SIS1‐SIS1*; left half) or a plasmid expressing Droj1 (*GPD‐DROJ1*; right half) in place of Sis1 were passaged onto rich medium (left columns). Cells were then transformed with a plasmid overexpressing Hsp104 (*GPD‐HSP104*) that normally cures [*PSI* ^+^] (right columns). Color phenotype assays are shown for representative transformants (*n* ≥ 12 for each strain). B. Lysates of representative cells from the right side of panel A were resolved by SDD‐AGE and subjected to immunoblot analysis using an antibody specific to Sup35. C. Lysates of representative cells from panel B were resolved by SDS‐PAGE and subjected to immunoblot analysis using an antibody specific to Hsp104. Load control shown is a nonspecific protein cross‐reacting with our Hsp104 primary antibody.

Because Droj1 is capable of supporting strong [*PSI^+^*] variants, we next questioned whether Droj1 is able to substitute for Sis1 in Hsp104‐mediated curing of these variants. The four strong [*PSI^+^*]‐bearing strains from Fig. 2 were transformed with a multicopy plasmid overexpressing Hsp104 (*GPD‐HSP104*) which normally results in rapid curing of multiple strong [*PSI* ^+^] variants in both backgrounds (Sporn and Hines, 2015). Transformants (*n* ≥ 12) were plated onto medium selective for the Hsp104 overexpression plasmid and subsequently onto rich medium to check for prion maintenance in these cells; Droj1 was deficient in replacing Sis1 in Hsp104‐mediated [*PSI^+^*] curing, as 12/12 colonies maintained [*PSI^+^*] in all four strains tested (Fig. 3A). Next, we subjected lysates of representative transformants to SDD‐AGE immunoblot analysis; as expected we found that all cells shown in Fig. 3A maintained [*PSI^+^*] as the color phenotype indicated (Fig. 3B). Finally, to eliminate the hypothesis that Droj1 expression alters Hsp104 curing by preventing cells from overexpressing Hsp104, lysates of representative transformants were subjected to SDS‐PAGE immunoblot analysis and visualized using an antibody recognizing Hsp104. We found that relative to all four initial strains, [*PSI^+^*] cells containing the *GPD‐HSP104* plasmid express Hsp104 at levels significantly above wild‐type expression (Fig. 3C).

### Requirements for Sis1 function in Hsp104‐mediated curing are variant dependent

**Figure 4:**
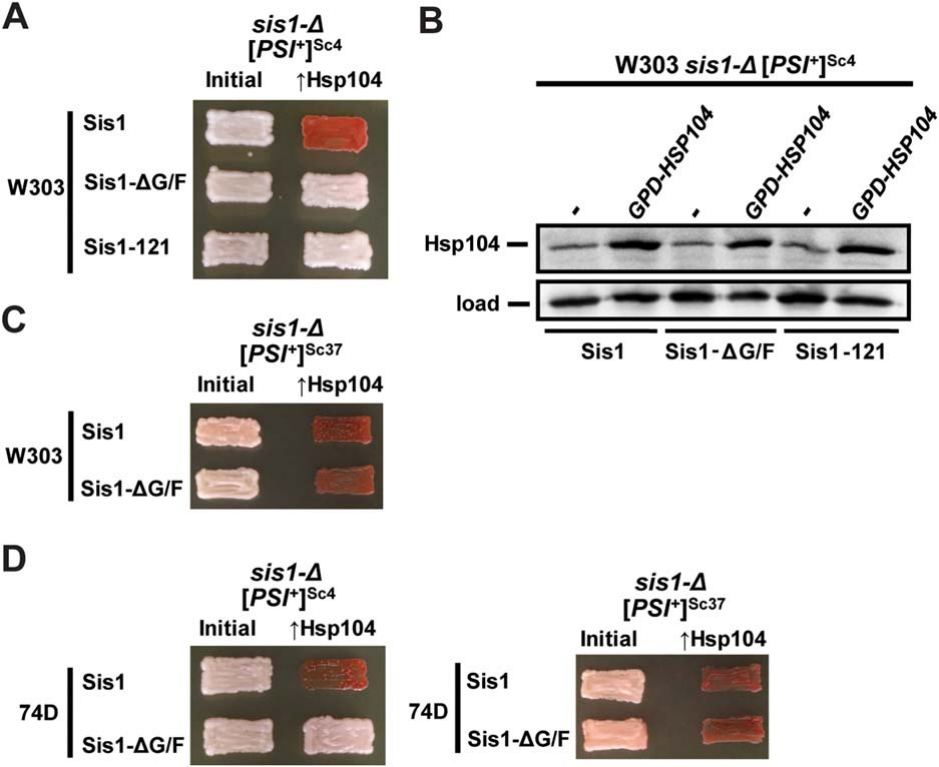
Sis1 domain requirements for Hsp104‐mediated curing of strong and weak [*PSI* ^+^] variants. A. Strong [*PSI* ^+^]^Sc4^ cells of the W303 genetic background lacking genomic Sis1 but expressing Sis1 from a *URA3*‐marked plasmid were transformed with plasmids expressing wild‐type Sis1 or Sis1 truncations Sis1‐ΔG/F or Sis1–121 (left column). Following loss of the *URA3*‐marked plasmid, cells were then transformed with a plasmid overexpressing Hsp104 (*GPD‐HSP104*) that normally cures [*PSI* ^+^]. Color phenotype assays are shown for representative transformants (*n* ≥ 10 for each variant). B. Lysates of strains lacking wild‐type Sis1 expression (*sis1*‐Δ) and expressing Sis1, Sis1‐ΔG/F or Sis1–121 from plasmids were resolved by SDS‐PAGE and subjected to immunoblot analysis using an antibody specific for Hsp104. Antibody specific for Ssc1 was used as a loading control. C. Same as panel A, but cells have the weak variant [*PSI* ^+^]^Sc37^, and Sis1–121 is omitted because it is unable to propagate this variant. D. Same as panels A and C, but cells are derived from the 74D‐694 genetic background.

Because Droj1 and Hdj1 maintain only strong [*PSI^+^*] variants, our investigations described above regarding Hsp104‐mediated curing did not address potential differences between the curing of weak vs. strong [*PSI^+^*] variants. Likewise, previous investigations of J‐protein function in this process never addressed the importance of [*PSI^+^*] variant strength, as only strong variants of [*PSI^+^*] were used. To investigate potential distinctions in Hsp104‐mediated [*PSI* ^+^] curing between weak and strong variants of [*PSI* ^+^] we first examined strains bearing the well‐studied strong variant [*PSI* ^+^]^Sc4^ and expressing commonly used Sis1 truncation mutants, Sis1ΔG/F and Sis1–121, that stably propagate [*PSI* ^+^] in place of the wild‐type protein (Kirkland *et al*., 2011; Harris *et al*., 2014; Stein and True, 2014bb). Sis1ΔG/F lacks only the glycine/phenylalanine (G/F) region whereas Sis1–121 consists of only the J‐domain and G/F region (Fig. 1). After transformation with *GPD‐HSP104*, [*PSI* ^+^]^Sc4^ was promptly cured (*n* = 10) in our wild‐type control strain (Fig. 4A). In sharp contrast, when a Sis1 truncation was expressed as the sole copy of Sis1, Hsp104‐mediated curing was either completely blocked (Sis1–121, 0 of 16 transformants cured) or drastically inhibited (Sis1‐ΔG/F, 2 of 17 transformants cured) (Fig. 4A). To rule out the hypothesis that these Sis1 truncations might simply affect Hsp104 expression directly, we examined the expression of Hsp104 in these strains before and after transformation with *GPD‐HSP104*. As expected, we found no distinct differences in the amount of Hsp104 expressed between the wild‐type control and Sis1 truncation strains that could explain these results (Fig. 4B).

Sis1–121 cannot propagate weak [*PSI* ^+^] variants in place of wild‐type Sis1 (Harris *et al*., 2014), but Sis1‐ΔG/F is capable of supporting weak [*PSI* ^+^] propagation (Higurashi *et al*., 2008; Harris *et al*., 2014). As such, we were able to use the latter construct to compare whether deletion of the G/F region of Sis1 has the same effect on Hsp104‐mediated curing of a weak [*PSI* ^+^] variant ([*PSI* ^+^]^Sc37^). In contrast to the strong variant, we found that [*PSI* ^+^]^Sc37^ was efficiently cured in every case (*n* = 12) in these strains (Fig. 4C). Finally, again using cell lines created in a previous investigation (Harris *et al*., 2014), we were able to reexamine both prion variants in the 74D‐694 genetic background. For both prion variants, the results were the same as those observed in the W303 background: for cells expressing Sis1‐ΔG/F and overexpressing Hsp104, 26 of 26 transformants maintained [*PSI* ^+^]^Sc4^ whereas 14 of 14 were cured of [*PSI* ^+^]^Sc37^, indicating that yeast genetic background is not a likely factor determining the different behaviors of the weak and strong prion variants observed here (Fig. 4D). Taken together, these results indicate that weak [*PSI* ^+^] variants have different requirements for J‐protein function in Hsp104‐mediated prion elimination relative to strong variants.

### Twelve cytosolic J‐proteins are dispensable for propagation, and Hsp104‐mediated curing, of two weak [*PSI* ^+^] variants

**Figure 5:**
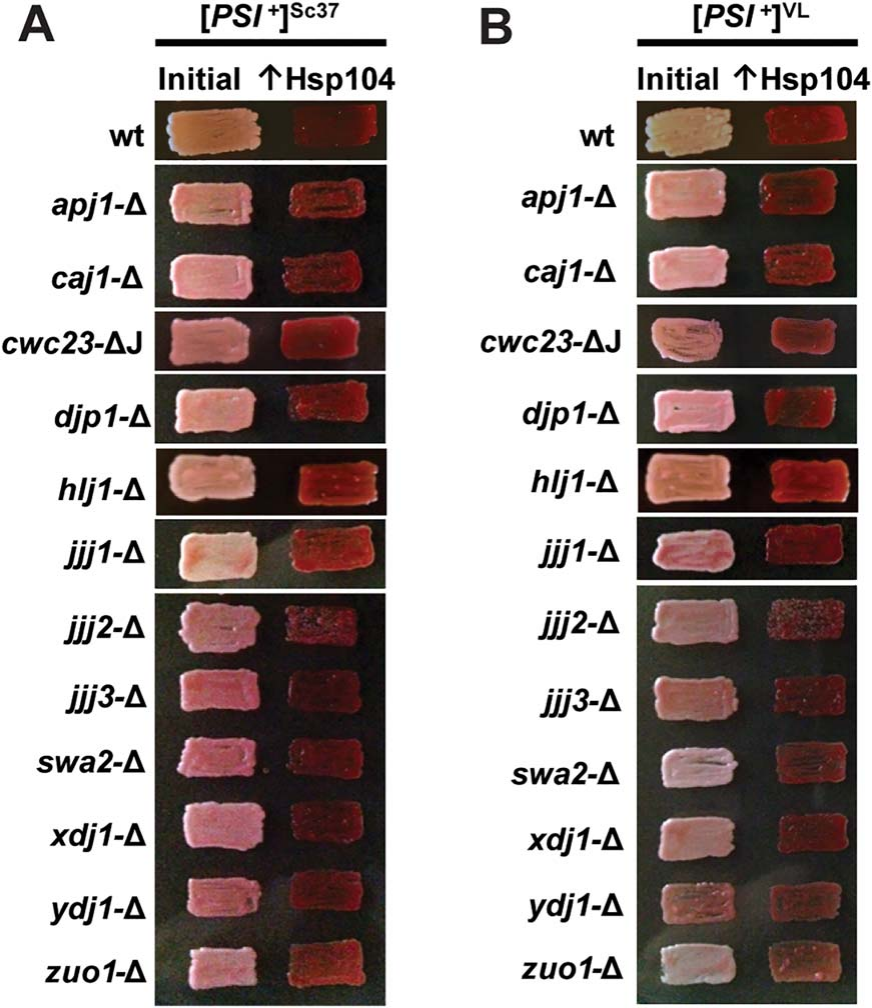
No cytosolic J‐protein other than Sis1 is required for propagation or Hsp104‐mediated curing of two weak [*PSI* ^+^] variants. Cells of the W303 genetic background were used which harbor either the weak [*PSI* ^+^] variant [*PSI* ^+^]^Sc37^ (A), or the weak variant [*PSI* ^+^]^VL^ (B). Weak [*PSI* ^+^] bearing cells lacking individual J‐proteins were passaged onto rich medium (left columns). Cells were then transformed with a plasmid overexpressing Hsp104 (*GPD‐HSP104*) that normally cures [*PSI* ^+^] (right columns). Color phenotype assays are shown for representative transformants (*n* ≥ 10 for each variant).

Although we and others have shown that Sis1 is required for the curing of strong [*PSI* ^+^] variants by overexpression of Hsp104 (Kryndushkin *et al*., 2011; Kirkland *et al*., 2011; Sporn and Hines, 2015), 12 other J‐proteins at least partially inhabit the yeast cytosol and of these, three (Apj1, Ydj1 and Swa2) have been previously implicated in prion biochemistry (Bradley *et al*., 2002; Kryndushkin *et al*., 2002; Lian *et al*., 2007; Hines *et al*., 2011a; Hines and Craig, 2011; Troisi *et al*., 2015; Oliver *et al*., 2017; Verma *et al*., 2017; Killian and Hines, 2018). We hypothesized that perhaps J‐proteins other than Sis1 may be required for Hsp104‐mediated curing of weak [*PSI* ^+^] variants. For example, we recently proposed that the J‐protein Swa2 may cooperate with Cpr7 in the propagation of [*URE3*], and Cpr7 has been shown to be important for the curing of strong [*PSI* ^+^] variants, which lead us to hypothesize that Swa2 could also be involved in this process (Kumar *et al*., 2015; Oliver *et al*., 2017). However, in order to address this and other possible roles for J‐proteins in Hsp104 curing, we first had to determine whether any cytosolic J‐protein other than Sis1 is essential for weak [*PSI* ^+^] propagation. In a previous study we ruled out essential roles for these J‐proteins, but only utilized a single strong variant of [*PSI* ^+^] (Higurashi *et al*., 2008), leaving open the possibility that weak [*PSI* ^+^] variants may exhibit ‘secondary’ J‐protein requirements in addition to Sis1, as we have recently found to be the case for the prions [*URE3*] and [*SWI* ^+^] (Hines *et al*., 2011b; Troisi *et al*., 2015; Oliver *et al*., 2017; Killian and Hines, 2018). To do this, we first chose to examine the weak variant [*PSI* ^+^]^Sc37^. We mated a [*PSI* ^+^]^Sc37^ strain with a set of 12 strains, each bearing a single J‐protein gene deletion (Sahi and Craig, 2007; Higurashi *et al*., 2008). In every case, following sporulation and tetrad dissection, we were easily able to isolate haploid F1 progeny with each J‐protein deletion that stably propagated the prion (Fig. 5A, left column). These results demonstrated for the first time that no cytoplasmic J‐protein other than Sis1 is essential for the propagation of a weak [*PSI* ^+^] variant and allowed us to subsequently assay for the potential requirement of any of these J‐proteins in Hsp104‐mediated [*PSI* ^+^] elimination. We next transformed each of these 12 strains with *GPD‐HSP104*; in every case [*PSI* ^+^] was completely eliminated in the resulting transformants (*n* ≥ 10), indicating that none of these J‐proteins are required for the curing of this variant by Hsp104 overexpression (Fig. 5A, right column). To ensure that these results are not specific to just one weak variant, we next replicated all of the same 12 genetic crosses and Hsp104 curing experiments to test both prion propagation and curing by Hsp104‐overexpression using a second weak variant, [*PSI* ^+^]^VL^. As we found with [*PSI* ^+^]^Sc37^, no J‐protein other than Sis1 is necessary for either propagation or Hsp104‐mediated curing of [*PSI* ^+^]^VL^ (Fig. 5B).

### ‘Antiprion DnaJ’ (Apj1) is critical for efficient elimination of strong [*PSI* ^+^]^STR^ by Hsp104 overexpression

**Figure 6:**
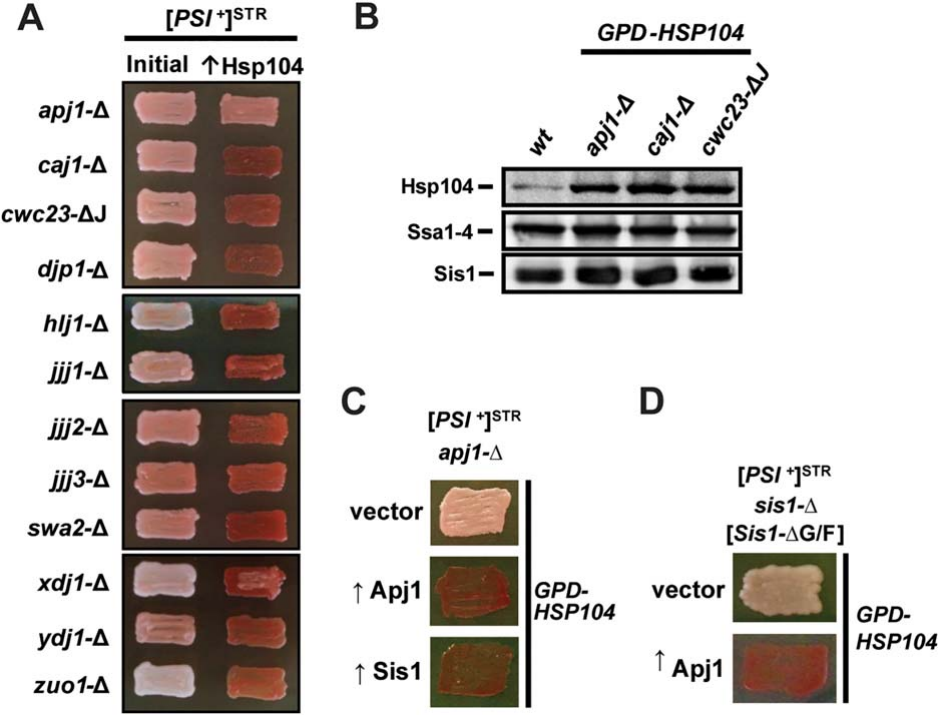
Lack of Apj1 expression, but not any of 11 other cytosolic J‐proteins, impairs Hsp104 curing of strong [*PSI* ^+^]^*STR*^. A. Strong [*PSI* ^+^]^*STR*^ bearing cells of the W303 genetic background lacking individual J‐proteins were passaged onto rich medium (left columns). Cells were then transformed with a plasmid overexpressing Hsp104 (*GPD‐HSP104*) that normally cures [*PSI* ^+^] (right columns). Color phenotype assays are shown for representative transformants: for *apj1*‐Δ 48 out of 90 transformants remained [*PSI* ^+^]; for all other strains curing was complete with *n* ≥ 10. B. Lysates of a wild‐type strain (*wt*), a strain lacking Apj1 expression (*apj1*‐Δ), a strain lacking Caj1 expression (*caj1*‐Δ) and a strain lacking the J‐domain of Cwc23 (*cwc23*‐ΔJ) were resolved by SDS‐PAGE and subjected to immunoblot analysis using antibodies specific for Hsp104, Ssa1–4 or Sis1. C. [*PSI* ^+^]^STR^ cells with a deletion of the *APJ1* gene (*apj1*‐Δ) were transformed first by plasmids overexpressing one of the two J‐proteins (↑Apj1 or ↑Sis1) or empty vector (vector), followed by a subsequent transformation with plasmid overexpressing Hsp104 (*GPD‐HSP104*) that normally cures [*PSI* ^+^]. Color phenotype assays are shown for representative transformants (*n* ≥ 10 for each variant). D. *sis1*‐Δ cells bearing [*PSI* ^+^]^STR^ and expressing Sis1‐ΔG/F from a plasmid were transformed with either empty vector (top row) or plasmid overexpressing Apj1 (bottom row) and subsequently transformed with *GPD‐HSP104*.

Sis1 is required for Hsp104‐mediated curing of strong [*PSI* ^+^] variants. Although we did not find any evidence suggesting that any other J‐protein is required for the curing of weak variants, it is plausible that strong variants could have more stringent requirements for J‐protein function as our Sis1 domain experiments indicated, which might include a requirement for a second J‐protein to achieve curing. Serendipitously, the strains necessary to test this hypothesis were already created in a prior investigation, each harboring a single J‐protein gene deletion and the strong [*PSI* ^+^] variant [*PSI* ^+^]^STR^ which was previously shown to propagate stably in these strains (Higurashi *et al*., 2008). As the strains bearing weak [*PSI* ^+^] variants, following transformation with *GPD‐HSP104*, [*PSI* ^+^]^STR^ was efficiently eliminated in 11 of the 12 strains (*n* ≥ 8 transformants for each strain); surprisingly however, across numerous experimental attempts (*n* = 90 total transformants), we found distinctive resistance to Hsp104 curing in the strain lacking the J‐protein Apj1, originally named ‘antiprion DnaJ’ because its overexpression cured a synthetic prion (Kryndushkin *et al*., 2002), with slightly more than half of all transformants (48/90) remaining [*PSI* ^+^] (Fig. 6A). Two additional rounds of re‐passaging on fresh medium selective for the Hsp104‐overexpression plasmid revealed no additional changes in prion status. It is unclear why some transformants ultimately resulted in cured populations while others did not, however others have similarly noted significant heterogeneity in cell populations undergoing Hsp104‐mediated curing (Reidy and Masison, 2010; Park *et al*., 2012; Ness *et al*., 2017; Zhao *et al*., 2017).

In order to rule out the possibility that deletion of *APJ1* might protect cells from Hsp104 overexpression through a stress response which might alter the expression of other proteins known to affect Hsp104 curing (i.e., elevate Ssa expression and lower either Hsp104 or Sis1 expression), we examined the expression of these proteins in several of our J‐protein deletion strains and in a wild‐type strain without Hsp104 overexpression. The amounts of Hsp104, Ssa and Sis1 in the *apj1*‐Δ strain were similar to those in other deletion strains in which curing occurred normally (Fig. 6B), indicating that the effect on Hsp104 curing due to the loss of Apj1 cannot be attributed to altered amounts of these proteins.

### Apj1 and Sis1 have overlapping functions in Hsp104‐mediated prion curing

To confirm that the loss of Apj1 is specifically responsible for the impairment of Hsp104 curing, rather than another unknown polymorphism in the strain, we next added back Apj1 by transforming our [*PSI* ^+^]^STR^, *apj1*‐Δ strain with a plasmid expressing Apj1. As expected, normal Hsp104 curing was restored; 20 of the 20 transformants were cured compared to 7 of the 20 cured for the *apj1*‐Δ control strain (Fig. 6C). Because both Apj1 and Sis1 are independently required for efficient curing by Hsp104 overexpression, we wondered if the two proteins might act through similar, and perhaps overlapping, mechanisms. If so, then overexpression of Sis1 might compensate for the loss of Apj1 in this process. Indeed, [*PSI* ^+^]^STR^ was efficiently cured (20/20 transformants) by *GPD‐HSP104* in a *apj1*‐Δ strain overexpressing Sis1 in place of Apj1 (Fig. 6C) indicating that the two proteins likely share overlapping functions.

Our results reported so far indicate that Sis1 and Apj1 promote Hsp104‐mediated curing of strong [*PSI* ^+^] and that overexpression of Sis1 can compensate for the loss of Apj1. To determine if this potential duplication of function is symmetrical or hierarchical, we asked if overexpression of Apj1 could compensate for Sis1 truncation. To do this, we transformed [*PSI* ^+^]^STR^ *sis1*‐Δ cells expressing Sis1‐ΔG/F from a plasmid with a second plasmid overexpressing Apj1. Importantly, propagation of the prion was unaffected by Apj1 overexpression. We next transformed this strain, along with the parental strain without Apj1 overexpression, with *GPD‐HSP104*. As previously observed, cells expressing only Sis1‐ΔG/F, without Apj1 overexpression, were protected from Hsp104 curing (0/20 transformants cured, Fig. 6D, top row) however overexpression of Apj1 partially restored Hsp104 curing (5/10 transformants cured, Fig. 6D, bottom row) indicating that Apj1 can at least partially substitute for Sis1 in this process.

**Figure 7:**
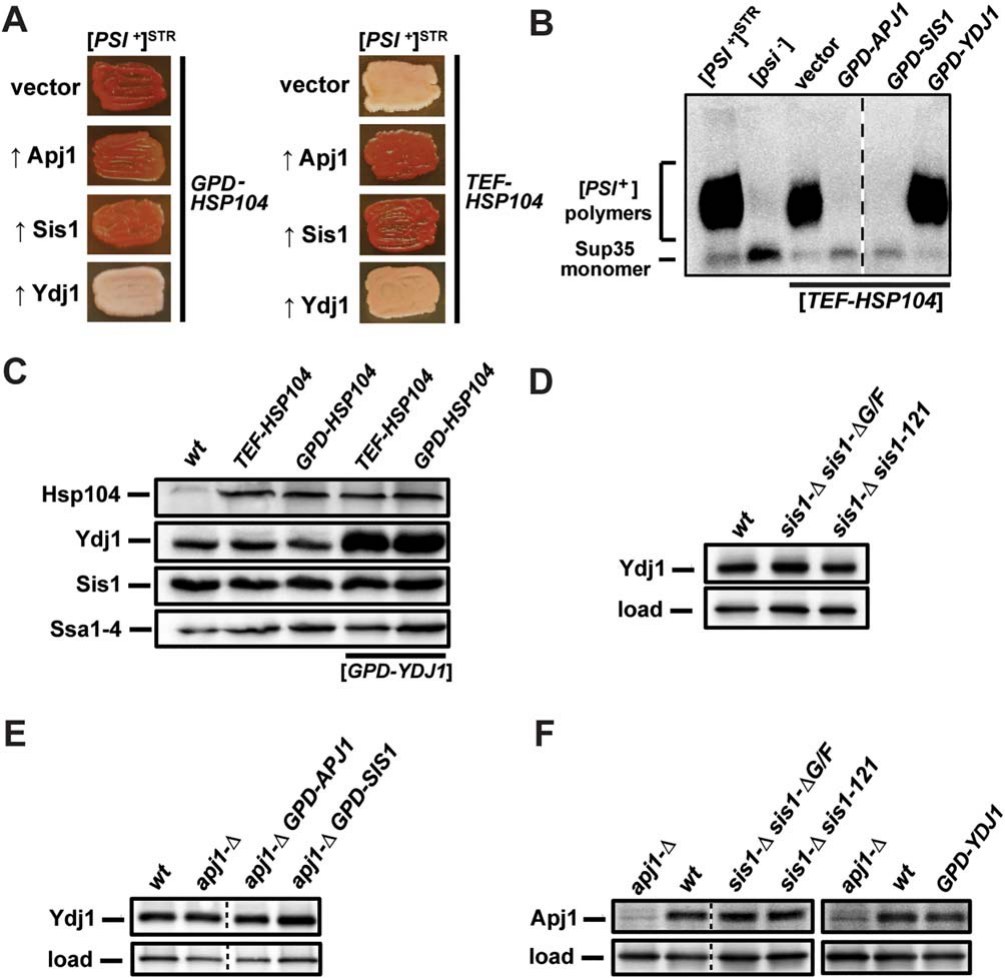
Ydj1 overexpression blocks Hsp104‐mediated curing of strong [*PSI* ^+^]^STR^ in a manner unrelated to changes in the expression of other relevant chaperones. A. [*PSI* ^+^]^STR^ cells were transformed first by empty vector or plasmids overexpressing various J‐proteins, followed by a subsequent transformation with plasmid overexpressing Hsp104 driven by either the *GPD* (left) or *TEF* (right), promoter. Color phenotype assays are shown for representative transformants (*n* ≥ 10). B. Lysates of representative cells from panel A were resolved by SDD‐AGE and subjected to immunoblot analysis using an antibody specific to Sup35. Dotted lines separate lanes taken from different parts of the same gel. C. Lysates of a wild‐type strain, or strains overexpressing Hsp104, or both Hsp104 and Ydj1, were resolved by SDS‐PAGE and subjected to immunoblot analysis using antibodies specific for Hsp104, Ydj1, Sis1 or Ssa1–4. D. Lysates of a wild‐type strain or *sis1‐Δ* cells containing plasmids expressing truncated Sis1 were resolved on SDS‐PAGE and subjected to immunoblot analysis using an antibody specific to Ydj1. Antibody specific for Ssc1 was used as a loading control. E. Lysates of a wild‐type strain and strains lacking Apj1 but overexpressing Sis1 or Apj1 were resolved and visualized as in panel D. F. Lysates of wild‐type and *apj1‐Δ* strains as well as strains expressing truncated versions of Sis1 from a plasmid in place of endogenous Sis1 or overexpressing Ydj1 were resolved on SDS‐PAGE and subjected to immunoblot analysis using an antibody specific to Apj1. Antibody specific for Ssc1 was used as a loading control.

As noted by others, overexpression of Sis1 accelerates Hsp104‐mediated curing of strong [*PSI* ^+^] (Kirkland *et al*., 2011; Kryndushkin *et al*., 2011). Given Apj1's apparently similar role in promoting curing, and normally low expression (Ghaemmaghami *et al*., 2003), we speculated that perhaps Apj1 overexpression may likewise enhance curing in otherwise normal strains. To test this, we first transformed cells harboring [*PSI* ^+^]^STR^ with plasmids overexpressing Sis1 or Apj1, or empty vector; [*PSI* ^+^]^STR^ was stably propagated in these strains in every case. Next, we transformed all cells with *GPD‐HSP104*; however, because the prion was eliminated within the period needed to select transformants and allow for color development, no difference in curing was observed: all cells, including the control, were completely cured upon first examination (Fig. 7A, left column). To circumvent this issue, we repeated the experiment using an alternative plasmid that expresses Hsp104 from the weaker *TEF* promoter (*TEF‐HSP104*) and reproducibly cures cell cultures of [*PSI* ^+^] less effectively than does the *GPD* plasmid. Using this system, upon first examination of transformants on rich media, wild‐type cells still showed mixtures of [*PSI* ^+^] and [*psi* ^–^] colonies whereas cells overexpressing either Sis1 or Apj1 cured completely with no phenotypically [*PSI* ^+^] colonies present (Fig. 7A, right column). These results confirm previous observations regarding Sis1 overexpression (Kirkland *et al*., 2011; Kryndushkin *et al*., 2011) and demonstrate that as Sis1, Apj1 when overexpressed can enhance Hsp104‐mediated prion curing. Overall these experiments reveal that overproduction of either Apj1 or Sis1 can enhance [*PSI* ^+^] curing and compensate for a deficiency of function in the other protein, indicating that the two proteins likely share a common functional role in Hsp104‐mediated curing.

### Apj1 and Ydj1 have reciprocal effects on Hsp104‐mediated elimination of strong [*PSI* ^+^]

Because overexpression of Apj1 and Sis1 had the same effect, we wondered if this enhancement of curing was simply due to an increase in generic J‐protein activity. To address this, we tested whether overexpression of Ydj1, the most abundant J‐protein in the cytosol (Sahi and Craig, 2007), would have a similar effect. The results of Ydj1 overexpression were both surprising and dramatic, as Ydj1 overexpression completely protected [*PSI* ^+^]^STR^ from Hsp104‐mediated curing driven by either *TEF‐HSP104* (0/20 transformants cured), or *GPD‐HSP104* (0/20 transformants cured, Fig. 7A). We subjected these same strains described above to SDD‐AGE analysis to ensure that the color phenotypes we observed accurately reflect the prion‐status of the cells in these experiments and to examine whether any changes in the aggregation state of Sup35 are occurring in cells protected from curing by Ydj1 overexpression. SDD‐AGE confirmed the prion status of all *GPD‐HSP104‐*transformed (*not shown*) and *TEF‐HSP104‐*transformed cells (Fig. 7B). Furthermore, no change in aggregate size was detectable for cells overexpressing Ydj1, despite the overexpression of Hsp104 in these cells (Fig. 7B), indicating that the effects of Hsp104 overexpression in these cells may be completely blocked by Ydj1 overexpression.

As with the effects of *APJ1* deletion, we wanted to discern if the protection afforded by Ydj1 overexpression might be due to altered expression of other proteins that affect Hsp104 curing. Again, this was not the case, as amounts of Hsp104, Ssa and Sis1 were similar between wild‐type cells and cells overexpressing Ydj1 (Fig. 7C), indicating that Ydj1 overexpression does not protect [*PSI* ^+^] from Hsp104 curing by altering the expression of these proteins.

If Ydj1 overexpression protects [*PSI* ^+^] from Hsp104 curing, then perhaps other J‐protein alterations which prevent curing do so by inducing an increase in Ydj1 expression. To address this possibility, we first returned to our Sis1 truncation strains to examine if Ydj1 protein expression is increased when Sis1 is mutated, as this would potentially account for the protection from curing afforded by those truncations. Although we did observe some variations in Ydj1 expression, these variations were small and did not correlate with Hsp104 curing (Fig. 7D), indicating that Sis1 alterations do not block Hsp104 curing by affecting endogenous Ydj1 protein expression. Likewise, we found no evidence for alterations in Ydj1 expression as a potential explanation for the lack of curing in cells lacking Apj1 (Fig. 7E). As a final consideration, we explored the converse possibility that Sis1 alteration and/or Ydj1 overexpression might adversely affect [*PSI* ^+^] curing by significantly decreasing the expression of Apj1. Once again, we found no evidence to suggest that this is the case (Fig. 7F). Thus, in summary, we conclude that the differences we observe in Hsp104 curing as a result of these J‐protein alterations are not due simply to a direct effect on the expression of these other chaperones.

### Reciprocal effects of Apj1 and Ydj1 are independent of the presence of [*RNQ* ^+^] and not specific to a single strong variant of [*PSI* ^+^]

**Figure 8:**
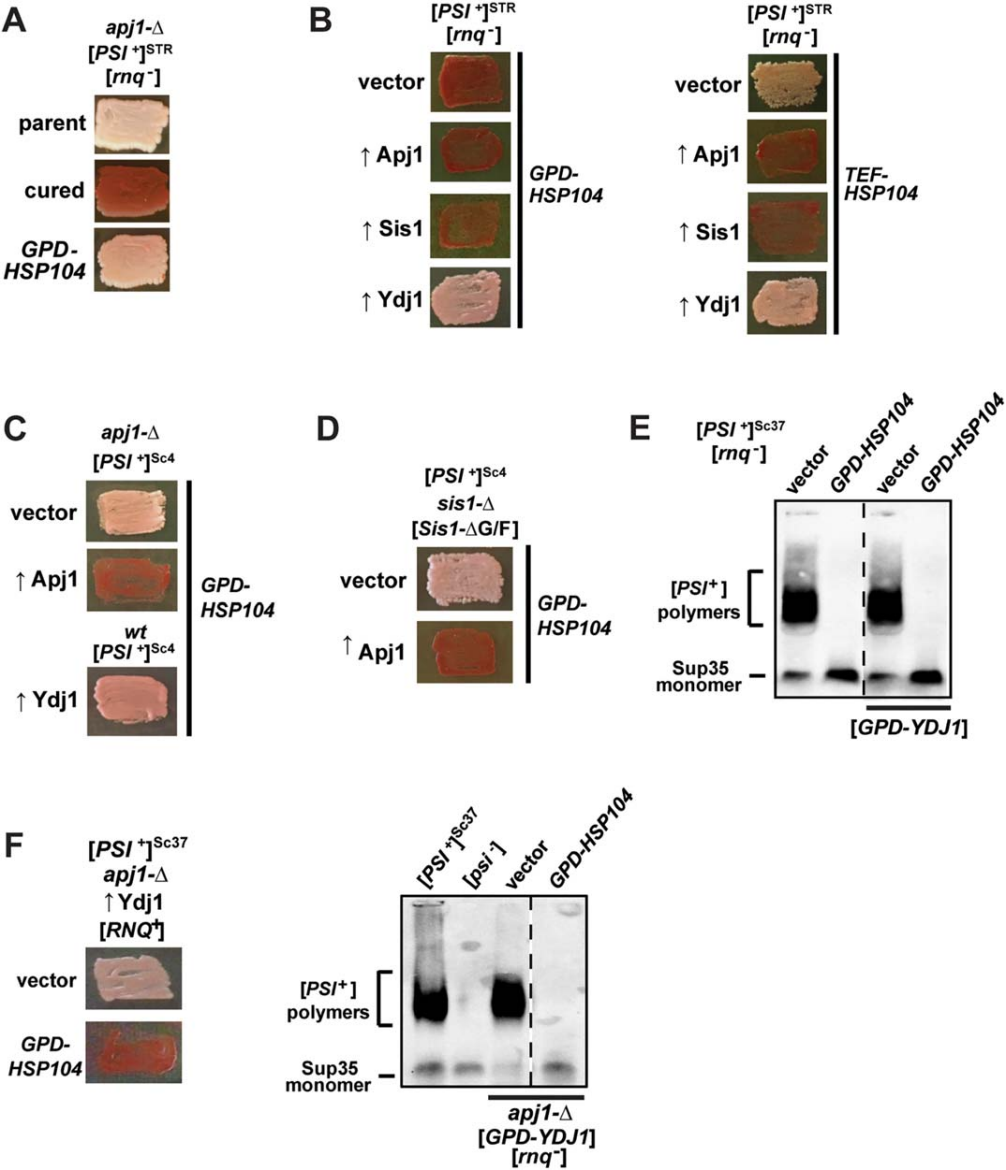
Effects of *APJ1* deletion and Apj1/Ydj1 overexpression on Hsp104‐mediated elimination are independent of [*RNQ* ^+^] but differ between strong and weak variants of [*PSI* ^+^]. Color phenotype assays or SDD‐AGE results are shown for representative transformants (*n* ≥ 10). A. Cells lacking *APJ1* and [*RNQ* ^+^] (denoted [*rnq* ^–^]) propagate [*PSI* ^+^]^*STR*^. These cells (parent) were treated with 4mM GdnHCl (cured) or transformed with a plasmid overexpressing Hsp104 (*GPD‐HSP104*) that normally cures [*PSI* ^+^]^STR^. B. [*rnq* ^–^]/[*PSI* ^+^]^STR^ cells were transformed first by empty vector or plasmids overexpressing various J‐proteins, followed by a subsequent transformation with plasmid overexpressing Hsp104 driven by either the *GPD* (left) or *TEF* (right), promoter. C. [*PSI* ^+^]^Sc4^ cells with a deletion of the *APJ1* gene (*apj1*‐Δ), or without (*wt*), were transformed first by either empty vector (*vector*) or a plasmid overexpressing either Apj1 (↑Apj1) or Ydj1 (↑Ydj1), followed by a subsequent transformation by a plasmid overexpressing Hsp104 (*GPD‐HSP104*) that normally cures [*PSI* ^+^]^Sc4^. D. *sis1*‐Δ cells bearing [*PSI* ^+^]^Sc4^ and expressing Sis1‐ΔG/F from a plasmid were transformed with either empty vector (*vector*) or plasmid overexpressing Apj1 (↑Apj1) and subsequently transformed with *GPD‐HSP104*. E. [*PSI* ^+^]^Sc37^ cells with or without vector overexpressing Ydj1 were transformed with empty vector or *GPD‐HSP104* and lysates resolved by SDD‐AGE and subjected to immunoblot analysis using an antibody specific to Sup35. Dotted lines separate lanes taken from different parts of the same gel. F. *apj1*‐Δ [*RNQ* ^+^]/[*PSI* ^+^]^Sc37^ cells overexpressing Ydj1 were transformed with empty vector or *GPD‐HSP104* (left side). *apj1*‐Δ [*rnq* ^–^]/[*PSI* ^+^]^Sc37^ cells overexpressing Ydj1 were transformed with empty vector or *GPD‐HSP104* and lysates resolved by SDD‐AGE and subjected to immunoblot analysis using an antibody specific to Sup35 (right side). Dotted lines separate lanes taken from different parts of the same gel.

Our initial experiments into the role of Sis1 (or its orthologs) in Hsp104‐mediated curing, both previously (Sporn and Hines, 2015), and here in Figs 2, 3, 4, utilized strains that were [*PSI* ^+^] and [*rnq* ^–^]. However, the J‐protein deletion strains used in experiments described in Figs 5 and 6 are originally from Sahi and Craig 2007 and are all derived from a [*RNQ* ^+^] strain (Sahi and Craig, 2007). Likewise, all previous experiments by others that addressed this specific topic were conducted in [*RNQ* ^+^] strains (Kirkland *et al*., 2011; Kryndushkin *et al*., 2011). Sis1 is known to strongly associate with [*RNQ* ^+^] aggregates in vivo (Sondheimer *et al*., 2001; Lopez *et al*., 2003), and sequestration of Sis1 by amyloids is also known to have significant impact on the amount of free Sis1 available for other purposes (Yang *et al*., 2013). As such, we considered whether the presence or absence of [*RNQ* ^+^] aggregates inside the cell may affect our experimental outcomes, particularly with the consideration that reduced amounts of free Sis1 might be expected to reduce the ability of Hsp104 to cure [*PSI* ^+^]. To address this issue, we systematically repeated our most important experiments in strains, which either possess or lack the [*RNQ* ^+^] prion as appropriate. We confirmed that deletion of *APJ1* does not impair propagation but does impede Hsp104‐mediated curing of strong [*PSI* ^+^] in a [*rnq* ^–^] strain (Fig. 8A), that overexpression of either Apj1 or Sis1 enhances Hsp104‐mediated curing of strong [*PSI* ^+^] in the absence of [*RNQ* ^+^] (Fig. 8B) and that Ydj1 overexpression completely blocks curing in a [*rnq* ^–^] background (Fig. 8B). Thus, none of the effects we have reported herein of various J‐proteins on Hsp104‐mediated curing are due to the presence or absence of [*RNQ* ^+^] in these strains.

Additionally, we wanted to confirm that both the pro‐ and anticuring effects of Apj1 and Ydj1, respectively, are not specific to the particular variant of [*PSI* ^+^] we have chosen for these experiments. To do this, we first crossed our *apj1*‐Δ strain to another strain bearing a different strong [*PSI* ^+^] variant, [*PSI* ^+^]^Sc4^. Following sporulation and tetrad dissection, we isolated haploid *apj1*‐Δ cells that stably propagate [*PSI* ^+^]^Sc4^; as [*PSI* ^+^]^STR^ (Higurashi *et al*., 2008), this variant also does not require Apj1 for stable propagation in yeast. Subsequent transformation with *GPD‐HSP104* failed to cure [*PSI* ^+^]^Sc4^ in the *apj1*‐Δ strain (0/10 transformants cured) compared to 10 of the 10 transformants cured in the wild‐type control strain expressing this variant. Further, overexpression of Apj1 in the same strain fully restored curing as expected, demonstrating that like [*PSI* ^+^]^STR^, efficient curing of [*PSI* ^+^]^Sc4^ by Hsp104 requires Apj1 (Fig. 8C). Next, to confirm that Ydj1 overexpression likewise protected this prion variant, we transformed our parental wild‐type [*PSI* ^+^]^Sc4^ with plasmid overexpressing Ydj1, followed by subsequent transformation with *GPD‐HSP104*. As observed for [*PSI* ^+^]^STR^, no curing occurred (10/10 transformants) when Ydj1 was overexpressed, despite Hsp104 overexpression (Fig. 8C). Finally, we again transformed [*PSI* ^+^]^Sc4^ *sis1*‐Δ [Sis1‐ΔG/F] cells with plasmid overexpressing Apj1 and subsequently transformed this strain, along with the parental strain without Apj1 overexpression, with *GPD‐HSP104*. As previously observed, cells expressing only Sis1‐ΔG/F, without Apj1 overexpression, were protected from Hsp104 curing (0/20 transformants cured) while overexpression of Apj1 partially restored Hsp104 curing (5/10 transformants cured, Fig. 8D). These results confirm that both the pro‐ and anticuring effects of Apj1 and Ydj1 on strong [*PSI* ^+^] variants are not specific to a single strong variant.

### Neither Ydj1 overexpression alone nor in combination with deletion of APJ1 protects weak [*PSI* ^+^] cells from Hsp104‐mediated curing

Either overexpression of Ydj1 or deletion of *APJ1* protects strong [*PSI* ^+^] cells from Hsp104 curing. Although deletion of *APJ1* did not protect weak [*PSI* ^+^] variants, we wondered if overexpression of Ydj1, alone or in combination with the loss of Apj1 expression, might afford a weak variant protection from Hsp104 curing as it does strong variants. This was particularly of interest because we found Ydj1 overexpression to be far more effective than deletion of *APJ1* in consistently blocking the curing of strong variants. To test this hypothesis, we first transformed *apj1*‐Δ and wild‐type strains bearing the weak variant [*PSI* ^+^]^Sc37^ with a plasmid overexpressing Ydj1. Importantly, and as expected, neither Ydj1 overexpression by itself (Fig. 8E), nor the combination of *APJ1* deletion and Ydj1 overexpression (Fig. 8F) affected [*PSI* ^+^]^Sc37^ propagation. Strikingly, subsequent transformation with *GPD‐HSP104* resulted in the complete curing (*n* ≥ 8) of [*PSI* ^+^]^Sc37^ cells overexpressing Ydj1, without deletion of *APJ1* (Fig. 8E) or with the deletion of *APJ1* in both [*RNQ* ^+^] and [*rnq* ^–^] strains (Fig. 8F). These results demonstrate that neither deletion of *APJ1* nor overexpression of Ydj1 has any observable effect on Hsp104 curing of this variant, either individually or in conjunction and regardless of [*RNQ* ^+^] status. Taken together with our other observations presented earlier, these results indicate that of the multiple J‐protein alterations that affect (positively or negatively) the curing of strong variants by Hsp104, none have any discernable effect on weak variants of [*PSI* ^+^].

## Discussion

### Sis1 involvement in prion propagation and Hsp104‐mediated curing

A summary of the key findings of this study is given in Table 1. To further examine the role of J‐proteins in Hsp104‐mediated curing, we utilized well‐characterized Sis1 constructs and examined multiple [*PSI* ^+^] variants. Similar experiments were recently conducted by Kirkland *et al*., but utilizing a single strong [*PSI^+^*] variant and yeast genetic background (Kirkland *et al*., 2011). Here, we found the Sis1 domain requirements for strong [*PSI^+^*] variants in Hsp104‐mediated curing to be consistent with those reported by Kirkland *et al*. (2011), which were characterized with a distinct strong [*PSI^+^*] variant in a different yeast genetic background. Our results confirm that loss of either the C‐terminal or glycine‐rich regions of Sis1 impairs curing, and in combination with the findings by Kirkland *et al*., suggest that both regions are likely involved in Hsp104‐mediated curing. From experiments with heterozygous diploids those authors noted that due to the recessive character of these mutants, the loss of curing is likely due to a loss of function in Sis1 rather than a disruption by the mutant in the function of another chaperone, and therefore, Sis1 is likely directly involved in this process (Kirkland *et al*., 2011). Recently, we also demonstrated that the human ortholog of Sis1, Hdj1, is capable of substituting for Sis1 in strong [*PSI* ^+^] variant propagation but not in Hsp104‐mediated curing (Sporn and Hines, 2015). Here, we found similar results for the *D. melanogaster* ortholog, Droj1. One notable observation from those experiments is that when Droj1 was the sole Sis1 ortholog expressed, aggregates resolved by SDD‐AGE shifted to higher molecular weights upon Hsp104 overexpression; this is congruent with observations we made in a prior investigation using Hdj1 (Sporn and Hines, 2015) and is similar to what others have observed while monitoring cells in the process of curing (Kryndushkin *et al*., 2003). These size shifts are noteworthy because they are observable in cells that are incapable of curing due to the presence of a deficient Sis1 ortholog. Therefore, they may provide a glimpse of an intermediate but arrested state in Hsp104 curing, in which, for reasons that still remain unclear, aggregates that are resolvable by SDD‐AGE increase in size prior to curing. Overall, these data indicate a partial conservation of function in eukaryotic evolution, as the functional properties of these metazoan orthologs are similar to those of truncated versions of yeast Sis1 and once again underscore the necessity of Sis1 in these processes.

**Table 1:** Summary of key findings.

Genetic modification		[*PSI* ^+^] propagation		Hsp104 curing	
Deletion	Overexpression	Strong [*PSI^+^*]	Weak [*PSI^+^*]	Strong [*PSI^+^*]	Weak [*PSI^+^*]
*sis1‐Δ*	*DROJ1*	Maintained	Lost	Not cured	–
*sis1‐Δ*	*sis1–121*	Maintained	Lost	Not cured	–
*sis1‐Δ*	*sis1‐ΔG/F*	Maintained	Maintained	Not cured	Cured
*sis1‐Δ*	*sis1‐ΔG/F*, *APJ1*	Maintained	–	Partially cured	–
11 cytosolic J‐proteins^a^	–	Maintained	Maintained	Cured	Cured
*apj1‐Δ*	–	Maintained	Maintained	Partially cured	Cured
*apj1‐Δ*	*SIS1*	Maintained	–	Cured	–
*apj1‐Δ*	*YDJ1*	–	Maintained	–	Cured
–	*SIS1*	Maintained	–	Increased curing	–
–	*APJ1*	Maintained	–	Increased curing	–
–	*YDJ1*	Maintained	Maintained	Not cured	Cured

The table summarizes the effects of various genetic modifications (gene deletions/protein overexpression) on the propagation and Hsp104‐mediated curing of strong and weak [*PSI* ^+^] variants as described in the *Results* section (see text for additional details).

**a.** Single deletions: *caj1‐Δ*, *cwc23‐Δj*, *djp1‐Δ*, *hlj1‐Δ*, *jjj1‐Δ*, *jjj2‐Δ*, *jjj3‐Δ*, *swa2‐Δ*, *xdj1‐Δ*, *ydj1‐Δ* and *zuo1‐Δ*.

In a prior investigation, one of us (JKH) and former coworkers demonstrated that none of the other 12 J‐proteins found at least partially in the *S. cerevisiae* cytosol are required for the propagation of either of two prions, [*RNQ* ^+^]^STR^ and [*PSI* ^+^]^STR^, however that investigation was limited to only those strong variants (Higurashi *et al*., 2008). Recent discoveries of secondary J‐protein requirements for the propagation of the prions [*URE3*] and [*SWI* ^+^] raise the question of whether these requirements are due to the relatively low propagon numbers per cell of these prions (Ripaud *et al*., 2003; Higurashi *et al*., 2008; Hines *et al*., 2011b; Hines and Craig, 2011). If so, then weak variants of other prions that also have low propagon numbers may share these requirements for prion propagation. Indeed, recent work has demonstrated significant variation of the J‐protein requirements among variants of the same prion (Harris *et al*., 2014; Stein and True, 2014a,b; Sporn and Hines, 2015; Killian and Hines, 2018). Our finding, that neither of two distinct weak [*PSI* ^+^] variants require a second J‐protein, negates this hypothesis. Rather, these results indicate that the requirement (or lack thereof) for additional chaperone action may be the result of more fundamental differences between prions and prion‐forming proteins apart from variation in the final amyloid structure, for example, sequence elements, or more likely amino acid composition of the prion‐forming protein, as we have suggested before (Hines and Craig, 2011; Killian and Hines, 2018).

### Potential roles for Sis1 and Apj1 in Hsp104‐mediated curing of strong [*PSI* ^+^] variants

No prior studies have examined the potential for J‐protein involvement in Hsp104‐mediated curing beyond just Sis1 and Ydj1. Here, our comprehensive screen, including all 13 members of the yeast cytosolic/nuclear J‐protein complement, uncovered significant genetic evidence for a role of Apj1 in this process. Apj1 has been implicated several times in the context of amyloid‐related protein‐misfolding in yeast (Kryndushkin *et al*., 2002; Willingham *et al*., 2003; Hines and Craig, 2011; Hines *et al*., 2011b). It was originally identified in a screen for cellular factors that interfered with the propagation of the synthetic yeast prion [ ${\text{PSI}}_{\text{PS}}^{+}$] when overexpressed, gaining the name Apj1 for ‘antiprion DnaJ’ (Kryndushkin *et al*., 2002). Apj1 was later identified in a screen for genes that when deleted enhance the toxicity of mutant Huntington (Willingham *et al*., 2003) and was implicated in the propagation of the prion [*SWI* ^+^] (Hines *et al*., 2011b, Hines and Craig, 2011). Owing in part to high sequence identity with Ydj1, Apj1 has sometimes been shown to act similarly to Ydj1 (Hines *et al*., 2011b; Gillies *et al*., 2012), with the most relevant example being the ability to rescue [*SWI* ^+^] in a strain lacking Ydj1 (Hines *et al*., 2011b). This partial overlap in functionality likely stems from the relatively recent emergence of Apj1 as the result of a *YDJ1* gene duplication event in Ascomycota (Sahi *et al*., 2013). Thus, there is significant overlap in overall structure between the two proteins: Apj1's domain structure (Fig. 1A) and location of key hydrophobic residues in its peptide‐binding domain are similar to that of Ydj1 (Sahi *et al*., 2013). Despite their similarities, here we found strikingly different effects of alterations in the expression of the two proteins. Likely structural explanations are that Apj1's glycine‐rich region and dimerization domain are elongated when compared to those of Ydj1 and, more notably, Apj1 and Ydj1 have dissimilar residue identities in the peptide‐binding cleft, indicating that Apj1 may bind a different, though not entirely distinct, set of client proteins than Ydj1 (Sahi *et al*., 2013). In contrast, our results strongly indicated that Apj1 and Sis1 have overlapping functions in Hsp104‐mediated prion curing as either protein, when overexpressed, could compensate for the lack of the other. These findings raise the following questions: what is the mechanism of Hsp104 curing, and what are the roles of Sis1 and Apj1 in that mechanism?

Of the multiple models for Hsp104‐mediated curing that have been proposed, two have been significantly debated in the recent literature: malpartitioning of [*PSI* ^+^] aggregates during cell division (Ness *et al*., 2017; Cox and Tuite, 2018; Matveenko *et al*., 2018) and the trimming of prion aggregates followed by eventual destruction of prion cores (Park *et al*., 2014; Zhao *et al*., 2017; Greene *et al*., 2018). In our opinion, our data reported here do not significantly support one model over the other, largely because neither model (nor previously favored models for Hsp104 curing) explicitly addresses the requirement for J‐proteins in the process. What could be the relevant function of Apj1 in either of these models? One plausible answer is that Apj1 may affect Hsp104‐curing by altering the degradation of sumoylated proteins. *APJ1* was indicated in a screen for synthetic genetic interactions with a deletion of *SLX5*, which encodes a subunit of a SUMO‐targeted ubiquitin ligase (Pan *et al*., 2006). Furthermore, in cells challenged by a reduction in sumo‐dependent ubiquitin ligase activity, loss of Apj1 exacerbated defects in the degradation of sumoylated proteins, consistent with Apj1's role in the proteolysis of proteins targeted for degradation by sumoylation (Sahi *et al*., 2013). This newly uncovered Apj1 function required both a functional J‐domain and C‐terminal peptide binding domain, indicating that Apj1 likely acts through Hsp70 in this process and that client‐protein binding is likely important. Apj1 is localized in mitochondria to a greater extent than other J‐proteins (Ghaemmaghami *et al*., 2003), and with an estimated 100 copies per cell, Apj1 is also the least abundant cytosolic J‐protein, especially relative to 90,000 copies per cell of Ydj1 (Ghaemmaghami *et al*., 2003; Gillies *et al*., 2012; Sahi *et al*., 2013). Yet despite these gross differences in expression level and localization, overexpression of Ydj1 was incapable of compensating this SUMO‐related functionality of Apj1 (Sahi *et al*., 2013), which aligns with our observations here regarding Hsp104 curing. There is also substantial evidence showing that Hsp104‐mediated curing involves the ubiquitin–proteasome system (Chernoff *et al*., 1999; Chernova *et al*., 2003; Allen *et al*., 2007; Reidy and Masison, 2010).

Both trimming and malpartitioning models are intriguing because a potential role for Apj1 is apparent in either. Briefly, Park *et al*. (2014) suggest that, independent of Hsp104's role in fragmenting prion aggregates, the protein also has a ‘trimming’ activity whereby it trims lengthy prion aggregates, removing Sup35 monomers before the remaining prion core is presented to the proteasome for degradation. It is plausible that Apj1 plays a role in promoting degradation of trimmed prion cores by the proteasome. Presently, it is unclear if the trimming activity of Hsp104, observed using the GFP‐tagged NGMC construct utilized by Greene and coworkers, acts on native Sup35/[*PSI* ^+^] aggregates – a key point to be resolved moving forward. The malpartitioning model, in contrast, proposes that Hsp104 catalytically immobilizes [*PSI* ^+^] propagons in the mother cell, likely through association with cytoskeletal elements. Indeed, Hsp104 is known to function in actin dynamics, and significant evidence has been presented demonstrating the interplay between the actin network and [*PSI* ^+^] prion stability (Bailleul *et al*., 1999; Ganusova *et al*., 2006; Erjavec *et al*., 2007; Tessarz *et al*., 2009). Likewise, sumoylation is known to affect protein localization, and both actin and tubulin are sumoylated in vivo (Panse *et al*., 2004; Castillo‐Lluva *et al*., 2010; Gareau and Lima, 2010). It is plausible that sumoylation plays an important role in the immobilization of [*PSI* ^+^] aggregates that results in malpartitioning, which is disrupted by the loss of Apj1. Alternatively, Apj1 deletion could impact a downstream protein degradation process, although evidence that Sup35 itself is degraded during curing is conspicuously lacking (Allen *et al*., 2007). Interestingly, our finding that Sis1 can compensate for a lack of Apj1 would imply that if Apj1's role in Hsp104‐mediated curing involves sumoylation, then Sis1 should be able to compensate. Thus, this model would predict that Sis1 should be capable of at least partially rescuing the sumoylation defects caused by deletion of *APJ1*, a testable prediction for future work.

### Ydj1 overexpression blocks Hsp104‐mediated curing

Congruent with how Apj1 has carved out a distinct role in the yeast cytosol over the course of fungal evolution, we also found that Ydj1 acts in a reciprocal manner to Apj1 in Hsp104‐mediated curing, acting as a powerful antagonist. Notably, three prior investigations found either no effect or only very minor effects of Ydj1 overexpression on Hsp104‐curing of strong [*PSI* ^+^] (Kirkland *et al*., 2011; Kryndushkin *et al*., 2011; Kiktev *et al*., 2012). The obvious explanation for this discrepancy is that these studies all utilized constructs that likely produced lower amounts of Ydj1 in vivo than our multi‐copy *GPD* vector. Although we were able to rule out an indirect effect of Ydj1 overexpression on the expression levels of Apj1, Sis1, Ssa and Hsp104, it seems highly likely that due to partial functional overlap and the tremendous amount of Ydj1 molecules in the cell, Ydj1 may compete directly with Sis1, Apj1 or both when overexpressed. We note, however, that competition with Apj1 alone cannot account for our observations, as complete deletion of *APJ1* merely reduced Hsp104‐mediated curing whereas Ydj1 overexpression blocked it completely, suggesting that Ydj1 must do more than just block the action of Apj1. We favor a model in which Ydj1 competes with both Apj1 and Sis1, either directly or by redirecting Hsp70/Hsp104 to alternative cellular targets as was recently described (Reidy *et al*., 2014), effectively reducing the amount of Hsp104 available for prion elimination. However, despite our efforts to exclude potential alterations in the expression of multiple proteins, because our observations are made using live cells, we cannot fully exclude indirect effects of these chaperone alterations at this time.

Our data are also congruent with observations from Masison and co‐workers that indicate that the dominant mutant Ssa1–21 destabilizes [*PSI* ^+^] in a similar manner as Hsp104 overexpression (Hung and Masison, 2006; Reidy and Masison, 2010; Kirkland *et al*., 2011). Both processes require similar functions of Sis1, Hsp90 and Hsp90 co‐chaperones among other similarities (Reidy and Masison, 2010; Kirkland *et al*., 2011). Previously, it was reported that deletion of *YDJ1* enhanced the antiprion effects of Ssa1–21, indicating that Ydj1 had a protective effect on [*PSI* ^+^] (Jones and Masison, 2003), but their finding that Ydj1 had no protective effect against Hsp104 curing was an unexplainable discrepancy in this theory (Kirkland *et al*., 2011). Our finding of such an effect resolves this discrepancy and further supports the notion that these two curing mechanisms have numerous overlapping elements. Based on our observations, we would further speculate that impairment of Apj1 function may also suppress Ssa1–21.

### Differential J‐protein requirements for Hsp104‐mediated curing of distinct [*PSI* ^+^] variants

In sharp contrast to our observations using strong [*PSI* ^+^], which uncovered requirements for two J‐proteins and sensitivity to a third, our work revealed no evidence whatsoever for the need of J‐protein function for the curing of weak [*PSI* ^+^] variants, nor was the process sensitive to ectopic J‐protein overexpression. This included attempts to combine both the protective effects we observed with *APJ1* deletion and Ydj1 overexpression simultaneously, with no effect. Weak [*PSI* ^+^] variants are known to cure faster than strong variants upon Hsp104 overexpression; as such we expected that the requirement for J‐protein function may be less stringent for weak variants, since these variants may simply be easier to cure. Indeed, weak [*PSI* ^+^] variants differ from strong variants in both the structure and stability of the amyloid core as well as in the mobility of the M‐domain of Sup35 – a region thought to bind Hsp104 during Hsp104‐mediated curing, with weak fibers having more mobile residues in this region which may aid Hsp104 binding (Helsen and Glover, 2012a; Frederick *et al*., 2014; Tanaka *et al*., 2004, 2006; Toyama *et al*., 2007). Furthermore, one study described the following two types of Hsp104 binding sites on Sup35: one readily exchanges with the free pool of Hsp104 and is similar for both strong and weak variants and the other tightly binds Hsp104 and is more prevalent in a weak variant leading to more Hsp104 to be bound to weak than strong fibers (Frederick *et al*., 2014). Thus, one explanation for our findings would be a simple stringency model in which both strong and weak variants are cured via the same biochemical mechanism, but with a less stringent requirement for J‐protein activity (Sis1 or Apj1) for weak variant curing because of inherent differences in Hsp104 binding between the two variants. If this is the case, the reduced stringency for J‐protein activity in weak curing might simply be undetectable under our experimental conditions. Plausibly, native amounts of Sis1 and Apj1 present in the cytosol could compensate for a lack of function in the other protein. However, because Ydj1 overexpression blocks strong [*PSI* ^+^] curing completely, we would expect that overexpression of Ydj1 should still reveal these requirements. Even in a strain lacking *APJ1*, we found no effect whatsoever of Ydj1 overexpression, supporting the notion that the curing of weak [*PSI* ^+^] variants may occur by a biochemically distinct, J‐protein‐independent mechanism.

### Broader implications and future directions

Though the mechanism of Hsp104‐mediated curing continues to be debated, its importance as a protein quality control mechanism has broadened beyond the realm of ectopic expression. Wickner and coworkers recently showed that ostensibly the same mechanism is at work in yeast under normal cellular conditions, eliminating some variants of [*PSI* ^+^] as they arise; when this process was blocked using the same set of alterations that blocks Hsp104‐mediated curing, [*PSI* ^+^] formation rates went up as much as 10‐fold, giving rise to variants that were quickly eliminated once normal protein functions were restored (Gorkovskiy *et al*., 2017). Most relevant to note for our work was their finding that this process, as expected, was Sis1‐dependent. We would speculate on the basis of our new data that Apj1 overexpression would also promote that process, while Ydj1 overexpression or *APJ1* deletion might hinder it, impacting [*PSI* ^+^] formation rates and possibly changing the variant composition formed – these are testable hypotheses going forward.

**Figure 9:**
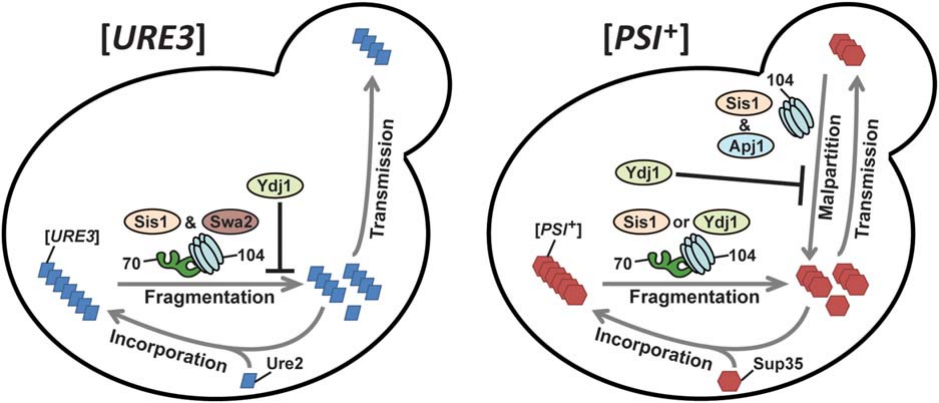
Model for J‐protein involvement in prion fragmentation and Hsp104‐mediated curing of strong [*PSI* ^+^] and [*URE3*]. Model comes from Matveenko *et al*. (2018) with modifications. Abbreviations: *70*, Hsp70; *104*, Hsp104. J‐proteins Sis1 and Swa2 are required for [*URE3*] propagation, presumably for aggregate fragmentation to produce propagons (left side) (Higurashi *et al*., 2008; Troisi *et al*., 2015). [*URE3*] is relatively insensitive to Hsp104‐mediated curing so this process is omitted (Kryndushkin *et al*., 2011). Ydj1 overexpression potently cures [*URE3*] via competition with Sis1 (Higurashi *et al*., 2008; Reidy *et al*., 2014). For strong variants of [*PSI* ^+^] (right side), Sis1 and plausibly Ydj1 can participate in aggregate fragmentation (Higurashi *et al*., 2008; Tipton *et al*., 2008; Kirkland *et al*., 2011). As shown in this work, Apj1 and Sis1 are required for Hsp104‐mediated curing (shown here as malpartitioning of propagons), which Ydj1 potently inhibits (see *Discussion* for additional details).

A recent review by Chernoff and coworkers considers both models of Hsp104‐mediated curing together with the notion of Hsp104‐mediated curing as an antiprion system, integrating those ideas with the considerable data that exists regarding the sensitivity of [*PSI* ^+^] and [*URE3*] to ectopic chaperone expression (Matveenko *et al*., 2018). Again, most relevant to this work was their focus on the impact of J‐proteins on these prions. We find their model compelling and can now add to it with our own contributions to bring a degree of symmetry to the model (Fig. 9, see Matveenko *et al*., 2018 for full model with alternative emphasis on the effects of Cur1, omitted here for clarity). In short, they proposed that [*PSI* ^+^] and [*URE3*] differ by their reciprocal sensitivities to two distinct J‐protein‐mediated processes that are always occurring: Hsp104‐mediated prion fragmentation to produce propagons and Hsp104‐mediated curing. For consistency with their model, we have used malpartitioning as our example for curing in the figure; however, the same ideas are equally applicable to trimming, and we agree that the two models are not necessarily mutually exclusive.

As shown in the model, [*URE3*] is exceedingly sensitive to reductions in Sis1 activity (Higurashi *et al*., 2008; Hines and Craig, 2011; Reidy *et al*., 2014). [*URE3*] is cured by overexpression of Ydj1 due to competition with Sis1 (Higurashi *et al*., 2008; Sharma *et al*., 2008; Reidy *et al*., 2014) but is largely insensitive to curing by Hsp104 overexpression (Kryndushkin *et al*., 2011; Barbitoff *et al*., 2017; Matveenko *et al*., 2018). The auxilin homolog Swa2 is also essential for [*URE3*] propagation (Troisi *et al*., 2015; Oliver *et al*., 2017), but its role in fragmentation specifically is speculative and independent from its strict reliance on Sis1 activity (Higurashi *et al*., 2008; Reidy *et al*., 2014). Thus, because [*URE3*] is relatively insensitive to Hsp104‐mediated curing, reductions in Sis1 activity provide no significant benefit but rather simply cause loss of [*URE3*] by affecting fragmentation (Matveenko *et al*., 2018). As such, Ydj1 is not expected to be protective when overexpressed, nor does deletion of *YDJ1* affect [*URE3*] (Troisi *et al*., 2015). In stark contrast, strong [*PSI* ^+^] variants are relatively insensitive to reductions in Sis1 activity (Higurashi *et al*., 2008; Kirkland *et al*., 2011; Harris *et al*., 2014; Reidy *et al*., 2014; Sporn and Hines, 2015). One of us (JKH) and former coworkers initially rationalized that this insensitivity may be due to the ability of another J‐protein to replace Sis1 in [*PSI* ^+^] aggregate fragmentation (Higurashi *et al*., 2008) and others have made the same assertion (Kirkland *et al*., 2011; Matveenko *et al*., 2018). Because Ydj1 is also found bound to [*PSI* ^+^] aggregates (Krzewska and Melki, 2006; Bagriantsev *et al*., 2008) it has been suggested as an alternative to Sis1 (Matveenko *et al*., 2018). However, its appearance in the model for [*PSI* ^+^] fragmentation is only speculative, as there is no data directly linking Ydj1 to [*PSI* ^+^] fragmentation. In contrast to [*URE3*], strong [*PSI* ^+^] variants are highly sensitive to Hsp104 curing, and as we have shown here, this process requires both Sis1 and Apj1 independently and is blocked by Ydj1. Thus, the model explains several observations: reductions in Sis1 activity reduce but do not eliminate [*PSI* ^+^] fragmentation (Higurashi *et al*., 2008) but also decrease [*PSI* ^+^] elimination by Hsp104, thereby reducing the negative impact on the prion. In contrast, Ydj1 overexpression does not negatively affect [*PSI* ^+^] because Ydj1 is nonessential for strong [*PSI* ^+^] propagation (Jones and Masison, 2003; Higurashi *et al*., 2008) and, in fact, enhances [*PSI* ^+^] (Barbitoff *et al*., 2017). This enhancement of [*PSI* ^+^] was attributed to the speculated role of Ydj1 in [*PSI* ^+^] propagon fragmentation (Matveenko *et al*., 2018), but we show here that this effect can be better explained by the reduction in Hsp104‐mediated [*PSI* ^+^] curing. This model then also explains the effects of alterations in Cur1 function on these two prions, as Cur1 re‐localizes Sis1 to the nucleus (Matveenko *et al*., 2018).

In investigating the role of J‐proteins in Hsp104‐mediated curing, we have further illuminated the functional diversity of the extraordinarily complex cytosolic J‐protein network of eukaryotes and expanded our knowledge base regarding prion–chaperone interactions in living cells, hopefully informing future efforts to utilize Hsp104 or other AAA+ ATPases. We were able to address two often overlooked aspects of this phenomenon: the role of J‐proteins and the impact of amyloid structural variation. Additional work will be necessary to understand why distinct prion variants have such drastically different requirements for Hsp104 curing, but perhaps the most significant question remaining unanswered involves the role of the Hsp70 Ssa in this process. Overexpression of Ssa inhibits Hsp104‐mediated curing, and curing has been strongly attributed to Hsp70‐independent binding to the M‐domain of Sup35 (Newnam *et al*., 1999; Helsen and Glover, 2012a,b; Winkler *et al*., 2012). Thus, the data present a bit of a paradox in that Ssa appears to be at best uninvolved and at worst antagonistic to curing, whereas its critical co‐chaperone Sis1 is required and can accelerate the process. As such, the role of Sis1 in this process has often been ignored in curing models due to the assumption that the process is Hsp70‐independent, as depicted in Fig. 9 and elsewhere (Park *et al*., 2014; Ness *et al*., 2017; Zhao *et al*., 2017; Cox and Tuite, 2018; Matveenko *et al*., 2018). Our findings here that not one but two Hsp70 co‐chaperones may be involved, with sensitivity to a third, bring this assumption further into question and necessitate a more nuanced approach for the consideration of the role of Hsp70 and its co‐chaperones in future models of this enigmatic amyloid‐clearing mechanism.

## Experimental procedures

### Yeast strains, plasmids and prion variants

Haploid *S. cerevisiae* W303 and 74D‐694‐derived strains were used throughout this investigation. W303 strains bearing [*PSI* ^+^]^STR^ and individual J‐protein gene deletions are from Higurashi *et al*. (2008). W303 and 74D [*PSI^+^*]/[*rnq* ^–^] strains used for all Sis1‐plasmid shuffling were those described in Harris *et al*. (2014). W303 strains Y1924, Y2054 and Y2461 (all [*rnq* ^–^], *trp1‐1*, *ura3‐1*, *leu2–3,112*, *his3–11,15* and *ade1–14*) were considered wild‐type strains bearing [*PSI* ^+^]^STR^, [*PSI* ^+^]^Sc4^ and [*PSI* ^+^]^Sc37^ respectively (Higurashi *et al*., 2008; Hines *et al*., 2011a).

To assay for weak [*PSI^+^*] maintenance in strains with individual deletions of 12 cytosolic J‐proteins, strains bearing each gene deletion and derived from PJ513a/Y639 ([*RNQ^+^*], [*psi^–^*], *trp1‐1*, *ura3‐1*, *leu2–3,112*, *his3–11,15*, *ade2‐1*, *can1–100*, *GAL2*, *met2‐1* and *lys2‐2*) were crossed with Y2461 ([*PSI* ^+^]^Sc37^) or Y2467 ([*PSI* ^+^]^VL^). To make a strain lacking *APJ1* but bearing both [*RNQ* ^+^] and [*PSI* ^+^]^Sc4^, Y2054 ([*RNQ* ^+^]/[*PSI* ^+^]^Sc4^) was crossed to strain Y1010 ([*RNQ* ^+^], *apj1*‐Δ) from Sahi and Craig (2007). To create *apj1*‐Δ strains bearing [*PSI* ^+^]^STR^ or [*PSI* ^+^]^Sc37^, but lacking [*RNQ* ^+^], Y1010 was cured of [*RNQ* ^+^] by treatment with GdnHCl and crossed to Y1924 ([*PSI* ^+^]^STR^/[*rnq* ^–^]) or Y2461 ([*PSI* ^+^]^Sc37^/[*rnq* ^–^]). Finally, to create a strain bearing both [*PSI* ^+^]^STR^ and [*RNQ* ^+^], Y1924 was crossed with Y639. Following all crosses, the resulting diploids were sporulated on potassium acetate minimal medium and subjected to tetrad dissection. The desired haploid strains were selected for by prototrophic growth on appropriate selective medium and the presence of prions was confirmed by color phenotype and/or SDD‐AGE as described below.

The various strong and weak [*PSI* ^+^] variants used in this work have distinct origins. The variant [*PSI* ^+^]^STR^ was originally induced by simultaneous overexpression of Sup35‐GFP and New1‐GFP (Osherovich and Weissman, 2001). [*PSI* ^+^]^VH^ and [*PSI* ^+^]^VL^ were created by overexpression of residues 1–114 of Pnm2 (Sup35^G58→D^) (King, 2001). Finally, [*PSI* ^+^]^Sc4^ and [*PSI* ^+^]^Sc37^ were created by polymerization of Sup35 in vitro, followed by transformation of [*psi* ^–^] cells with the resulting amyloid material, which then served to seed prion formation in vivo (Tanaka *et al*., 2004, 2006).

Plasmids used in this study are based on the pRS series (Mumberg *et al*., 1995). All plasmids were harvested from isolated DH5α *Escherichia coli* cells using QIAprep Spin Miniprep Kit (Qiagen, Valencia, CA). The following plasmids were used in this study: *p313‐SIS1‐SIS1* (Yan and Craig, 1999), *p424‐GPD‐SIS1* (Yan and Craig, 1999), *p324‐SIS1‐sis1‐ΔG/F* (Yan and Craig, 1999), *p324‐SIS1‐sis1–121* (Yan and Craig, 1999), *p424‐GPD‐YDJ1* (Higurashi *et al*., 2008), *p424‐GPD‐APJ1* (Hines *et al*., 2011b), *p424‐GPD‐DROJ1* (Lopez *et al*., 2003), *p416‐TEF‐HSP104*, *p413‐GPD‐HSP104* and *p416‐GPD‐HSP104* (Sporn and Hines, 2015).

### Plasmid shuffling and assays for prion loss

Plasmids bearing Sis1 constructs Sis1–121 and Sis1‐ΔG/F were used to transform plasmid shuffling strains and transformants were selected by growth on selective medium. Typically, at least 10 colonies were isolated in each experiment and plated onto medium containing 5‐fluoroorotic acid (5‐FOA), counter‐selecting against the *URA3*‐marked *SIS1* plasmid. *URA3* encodes orotidine 5‐monophosphate decarboxylase, which converts harmless 5‐FOA into cytotoxic 5‐fluorouracil (5‐FU), a potent inhibitor of thymidylate synthase; only cells which have lost the *URA3*‐marked *SIS1* plasmid survive to form colonies. Loss of the *URA3*‐marked plasmid was further confirmed in all cases by the failure to grow on synthetic medium lacking uracil. Shuffled cells were plated onto rich medium to detect for [*PSI^+^*] maintenance by colony color assay. [*psi* ^–^] colonies result in a block in adenine biosynthesis because of the lack of functional Ade1 enzyme and a buildup of the red‐pigmented intermediate in those cells. When Sup35 is aggregated into [*PSI* ^+^], colonies are white or pink due to restored adenine biosynthesis (Liebman and Chernoff, 2012). Prion maintenance was routinely further confirmed by plating cells onto rich medium (YEPD, Teknova) to test for colony color following growth in liquid medium containing 4 mM GdnHCl, which rapidly cures [*PSI* ^+^] (Eaglestone *et al*., 2000).

For experiments examining prion curing by Hsp104 overexpression, plasmids overexpressing Hsp104 were used to transform prion‐bearing strains and transformants were selected by growth on selective medium for 3 days at 30°C. Typically at least 10 colonies were isolated in each experiment and re‐plated 2–4 times onto selective medium at 30°C for an additional 2 days for each re‐plating to allow time for prion curing. Colonies were then plated onto solid, rich medium and allowed to grow at 25°C for 3–4 days to detect for [*PSI* ^+^] maintenance by colony color assay.

Semidenaturing detergent agarose gel electrophoresis (SDD‐AGE), a method for resolving detergent‐resistant aggregates, was used to routinely confirm the presence or absence of both [*RNQ* ^+^] and [*PSI* ^+^] and to determine relative size distributions of aggregates that enter the gel matrix (Bagriantsev *et al*., 2006). Briefly, 3–5 OD units of yeast cells were lysed using sterile glass beads by vortexing at 4°C with a Genie SI‐D248 Disruptor Shaker (Scientific Industries). Following centrifugation at 4°C, cleared lysates were mixed with SDS loading buffer and incubated at 25°C for 7 min. Aggregates were resolved in a 1.5% (wt/vol) tris‐glycine (0.1% SDS) agarose gel (SeaKem Gold PFGE agarose), and protein was transferred to a nitrocellulose membrane at 1 A for 1 h at 22°C in a tris‐glycine/methanol buffer. To visualize aggregates, membranes were blocked with 5% (wt/vol) milk and probed with antibodies specific for either Rnq1 or Sup35 (generous gifts from the Craig and Tuite labs respectively).

### SDS‐PAGE and immunoblot analysis

Protein extracts for SDS‐PAGE were prepared by harvesting 1.0 OD units of yeast cells followed by vortexing in 0.2 M NaOH at 25°C. Cells were spun at 14,500 r.p.m. on a table‐top centrifuge at 25°C and the supernatant was removed. Pellets were re‐suspended in a sample buffer containing SDS and boiled for 5 min before being resolved on a 10% polyacrylamide gel. The protein was transferred to a nitrocellulose membrane at 1 A for 1 h at 25°C in tris‐glycine/methanol buffer and probed with polyclonal antibodies raised against the following: Hsp104 (Cayman Chemicals, Ann Arbor, MI), Apj1, Sis1, Ssc1, Ssa1–4 and Ydj1 (Craig lab, University of Wisconsin‐Madison, Madison, WI).

## Conflicts of interest

The authors declare no competing interests.
